# Construction of chiral crown ethers in robust covalent organic frameworks for electrochromatographic enantioseparation

**DOI:** 10.1093/nsr/nwae256

**Published:** 2024-07-26

**Authors:** Shiguo Fu, Gaizhao Qin, Jinqiao Dong, Chen Yuan, Yan Liu, Li-Ming Yuan, Yong Cui

**Affiliations:** School of Chemistry and Chemical Engineering, Frontiers Science Center for Transformative Molecules and State Key Laboratory of Metal Matrix Composites, Shanghai Jiao Tong University, Shanghai 200240, China; Department of Chemistry, Yunnan Normal University, Kunming 650500, China; School of Chemistry and Chemical Engineering, Frontiers Science Center for Transformative Molecules and State Key Laboratory of Metal Matrix Composites, Shanghai Jiao Tong University, Shanghai 200240, China; School of Chemistry and Chemical Engineering, Frontiers Science Center for Transformative Molecules and State Key Laboratory of Metal Matrix Composites, Shanghai Jiao Tong University, Shanghai 200240, China; School of Chemistry and Chemical Engineering, Frontiers Science Center for Transformative Molecules and State Key Laboratory of Metal Matrix Composites, Shanghai Jiao Tong University, Shanghai 200240, China; Department of Chemistry, Yunnan Normal University, Kunming 650500, China; School of Chemistry and Chemical Engineering, Frontiers Science Center for Transformative Molecules and State Key Laboratory of Metal Matrix Composites, Shanghai Jiao Tong University, Shanghai 200240, China

**Keywords:** covalent organic framework, chiral material, porosity, capillary electrochromatography, enantioseparation

## Abstract

Capillary electrochromatography (CEC) is a rapidly emerging separation technique that merges the high separation efficiency of capillary electrophoresis with the exceptional selectivity of liquid chromatography. However, it remains a synthetic challenge to design functional chiral stationary phases (CSPs) with high chemical stability against acid and base in CEC enantioseparation. Here we demonstrate that incorporating chiral crown ethers into stable covalent organic frameworks (COFs) enables efficient and stable separations of racemates by CEC. This facilitates the crafting of two three-dimensional (3D) chiral COFs by polycondensation of a chiral 1,1'-binaphyl-20-crown-6-derived dialdehyde and tetraamines with diisopropyl substituents. Both feature an 11-fold interpenetrated diamond framework, characterized by tubular open channels decorated with chiral crown ethers serving as enantioselective recognition and binding sites. These frameworks demonstrate excellent stability in water, acid and base, thanks to the presence of bulky isopropyl groups that shield the dynamic imine linkages. Moreover, the precisely defined COF channels enhanced the accessibility of the enclosed crown ethers to the analytes while providing strong protection against harsh environments, rendering them suitable for CSPs in CEC separations. They can effectively separate some important enantiomers, including ketones, epoxides and alkaline substances, when utilized as coatings on chiral columns, particularly facilitating the chiral separation of drugs. This study advances the application of COFs in electrochromatographic separations, expanding the scope of porous materials design and engineering to create COFs with targeted enantioselective properties.

## Teaser

Two crown ether-decorated 3D CCOFs were designed and synthesized by imine condensations of tetrahedral tetraamines and an enantiopure 1,1'-binaphthyl-20-crown-6-derived dialdehyde. Both CCOFs exhibit high chemical stability against water, acid and base, serving as effective chiral stationary phases (CSPs) with exceptional selectivity for CEC-based separation of challenging enantiomers, especially racemic drugs.

## INTRODUCTION

The separation of enantiomers is of vital importance in chemistry and pharmacology due to the distinct biological and pharmacological effects of these compounds [1,2]. Among various enantioselective resolution techniques, chromatography, particularly high-performance liquid chromatography (HPLC) and gas chromatography (GC) over chiral stationary phases (CSPs), has emerged as the most attractive and practical method for separating and obtaining pure enantiomers [3,4]. However, capillary electrochromatography (CEC), blending the high efficiency of capillary electrophoresis with the selectivity of HPLC, is still evolving in its capability for chiral resolutions [5,6]. In CEC, the mobile phase typically comprises an acidic or alkaline aqueous buffer solution, requiring the CSPs to have high chemical stability against acid and base. Therefore, the chemical stability and functionalities of traditional CSP materials such as amino acid derivatives [7], crown ethers [8], saccharides [9,10], ionic liquids (ILs) and polymeric ILs (PILs) [11,12], as well as the newly emerging porous solids like metal-organic frameworks (MOFs) [13–15], metal-organic cages (MOCs) [16], porous organic polymers (POPs) [17,18] and covalent organic frameworks (COFs) [19–22], need to be improved [23]. Here, we demonstrate that the incorporation of chiral crown ethers into robust COFs enables efficient and durable separation of racemates by CEC.

COFs represent an innovative category of crystalline porous polymers crafted from organic monomers linked by covalent bonds [24–27], offering diverse structural and functional versatility applicable across various fields [28,29]. In particular, the potential applications of chiral COFs (CCOFs) extend beyond optoelectronics [30,31], catalysis [32–35] and sensing [24,36,37], to encompass chiral molecule separation [38,39], representing a significant advancement in analytical science. Indeed, CCOFs have shown promise as CSPs in both HPLC [40–43] and GC [40,41], facilitating the resolution of racemates [43]. However, recent investigations into imine-based COFs, the most common type of COF material, as CSPs for CEC separation have revealed obvious limitations in separation efficiency, selectivity and durability. These issues are likely attributed to the limited chemical stability of the imine COFs and the lack of strong chiral recognition sites that can effectively interact with enantiomers [19–21]. Inspiringly, our recent studies have demonstrated that introducing steric bulky groups on both sides of the imine linkages in COFs greatly increases the framework stability and robustness in water, acid and base solutions [33,44]. This enhancement is clearly favorable when utilized as CSPs in CEC applications. Crown ethers, as macrocyclic host molecules, are well known for their high stereoselectivity and affinity in binding protonated chiral molecules [45]. These inspire us to integrate the advantages and overcome the defects of COFs and crowns, thereby crafting new CSPs for CEC separation. Unlike the traditional matrix for immobilizing crown ethers, the *de novo* synthesized COFs provide continuous and confined open nanopores, providing an accessible high-surface-area interface for interactions between crown ethers and analytes [24,46,47].

**Scheme 1.**
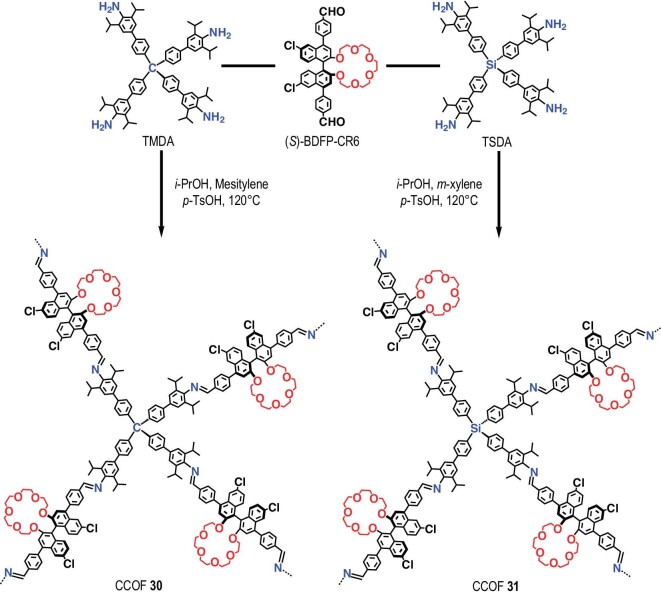
Synthesis of the two 3D CCOFs.

In this work, we design and synthesize two 3D CCOFs (denoted as 30 and 31) with high chemical stability against acid and base, by imine condensations of the custom-designed dialdehyde of enantiopure 2,2'-pentaethylene glycol-1,1′-binaphyl with tetraamines bearing diisopropyl substituents (Scheme 1). Both exhibit an 11-fold interpenetrated diamond structure, featuring crown ether moieties arranged periodically within accessible channels for guest molecules. Thanks to the abundant chiral microenvironments provided by crown ether-decorated COFs, we achieved baseline enantioseparation of a range of racemates, including three commonly used commercial drugs, using the CCOF-coated CEC columns. We further studied various factors, such as applied voltage, buffer solution concentration and acidity, to optimize the separation performance of racemates. The separation capacity of CCOFs was elucidated by density functional theory (DFT) calculations, revealing the diverse interactions between crown ether groups and racemates. This study thus validates the feasibility and practicality of using functional CCOFs as coatings in CEC for enantioseparation.

## RESULTS AND DISCUSSION

### Synthesis and characterization

As shown in Scheme 1, the enantiopure monomer (*S*)-BDFP-CR6, which contains crown ether groups, was synthesized by condensation of penta(ethylene glycol) di-*p*-toluenesulfonate and enantiopure dialdehyde derivatives of (*S*)-BINOL (see synthetic details in Supplementary Data). CCOFs **30** and **31** were synthesized through solvothermal reactions of (*S*)-BDFP-CR6 and tetraphenyl methane incorporating 2,6-diisopropyl aniline (TMDA) or tetraphenyl silane incorporating 2,6-diisopropyl aniline (TSDA) in *iso*-propanol, mesitylene (or *m*-xylene) and *p*-toluenesulfonic acid solution at 120°C. After 3 days, dark yellow crystalline solids were obtained in yields of ∼87% and ∼81%, respectively.

The as-prepared two CCOFs underwent thorough characterization using various spectroscopic techniques. Fourier transform infrared (FT-IR) spectra showed that a substantial weakening and almost negligible presence of the C=O stretching band (1701 cm^−1^) was observed in both materials, accompanied by the appearance of C=N bond stretching bands at 1634 cm^−1^ for CCOF **30** and 1633 cm^−1^ for CCOF **31**, indicating the successful polymerization process (Fig. S1 in Supplementary Data). The ^13^C cross-polarization magic-angle spinning (CP-MAS) nuclear magnetic resonance (NMR) signals of two COFs clearly matched the expected structure (Fig. S2). Specifically, the resonance at 161.9 ppm for CCOF **30** and 165.1 ppm for CCOF **31** ppm corresponded with the carbons of the imine groups, demonstrating the success of the Schiff-base reactions. As anticipated, circular dichroism (CD) spectra of the two CCOFs, synthesized from *R*- and *S*-enantiomers of BINOL dialdehyde derivatives, displayed mirror relationships and exhibited distinct Cotton effects, confirming their enantiomeric nature (Fig. S3).

**Figure 1.**
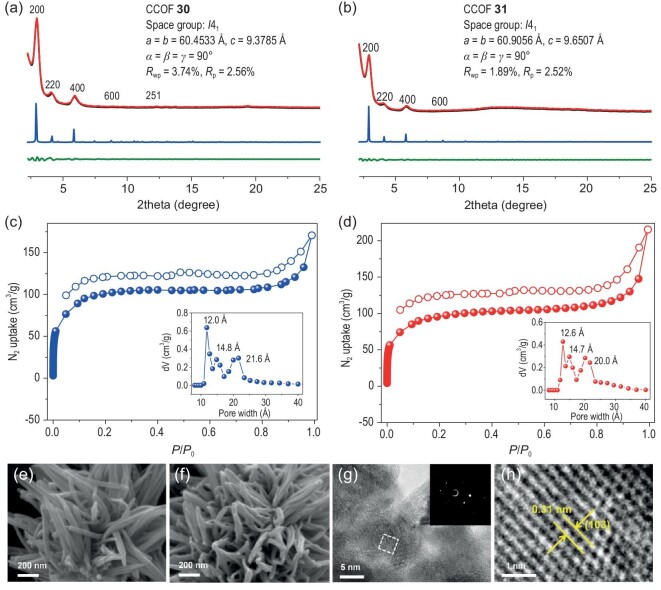
(a, b) PXRD patterns of the two CCOFs with experimental profiles in red, Pawley refined in black, calculated in blue, and the difference between the experimental and refined PXRD patterns in dark green. N_2_ adsorption-desorption isotherms (77 K) and pore size distribution profiles (inset) of (c) CCOF **30** and (d) **31**. SEM images of (e) CCOF **30** and (f) **31**. (g) HR-TEM image of CCOF **30** (inset: selected area electron diffraction pattern of CCOF **30**). (h) Magnified high-resolution lattice fringe image from the white dashed square in (c).

The successful formation of crystalline frameworks of CCOFs **30** and **31** was confirmed by powder X-ray diffraction (PXRD) (Fig. 1a and b). Based on previous reports and considering the molecular geometry of the monomers, several distinct structures were simulated and optimized utilizing the Materials Studio software package. The simulation results revealed that both CCOFs adopt an 11-fold interpenetrated ***dia*** topology with the chiral *I*4_1_ space group, respectively. In the case of CCOF **30**, the unit cell parameters were determined as follows: *a* = *b* = 60.4533 Å, *c* = 9.3785 Å, *α* = *β* = *γ* = 90°. Furthermore, the experimental PXRD pattern of CCOF **30** exhibiting (200), (220), (400), (600) and (251) facets at 3.05°, 4.20°, 6.02°, 8.92° and 12.31° respectively, corresponded well with the simulated model. The lattice model and Pawley refinement demonstrated good agreement factors (*R*_wp_ = 3.74% and *R*_p_ = 2.56%). For CCOF **31**, the experimental PXRD pattern displayed peaks at 2.91°, 4.04°, 5.85° and 8.73°, assigned to (200), (220), (400) and (600) facets (Fig. 1b), respectively, which also aligned with the simulated model. The Pawley refinement provided lattice parameters of *a* = *b* = 60.9056 Å, *c* = 9.6507 Å and *α* = *β* = *γ* = 90°, showing good agreement factors (*R*_wp_ = 1.89%, *R*_p_ = 2.52%). PXRD patterns were also calculated for both CCOFs with other structures, but these calculated patterns did not match well with the experimental patterns (Fig. S4).

**Figure 2.**
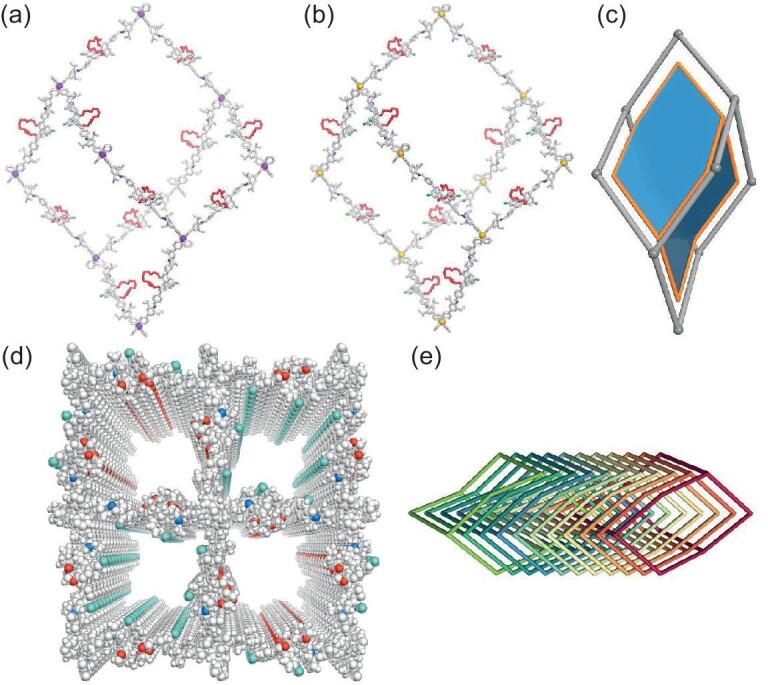
Structural representations of the two BINOL-based COFs. An adamantine-like cage in (a) CCOF **30** and (b) **31**. (c) The *dia* net model. (d) A space-filling model of the 3D structure of CCOFs **30** and **31** viewed along the *c* axis (only the channel size of CCOF **30** is shown for clarity). (e) 11-fold interpenetration of a diamond net. C, gray; N, blue; H, white; O, red; Cl, green; the central C in CCOF **30**, purple; the central Si in CCOF **31**, yellow.

Examination through scanning electron microscopy (SEM) and transmission electron microscopy (TEM) revealed a rod-like morphology for both CCOFs (Fig. 1e and f, and Fig. S5). In addition, we have performed high-resolution TEM (HR-TEM) to further confirm the crystallinity of CCOF **30** (Fig. 1g and h). Displaying a high-resolution lattice fringe image, the result showed that the periodicity of pore patterns correlates with the XRD refined structure. Consequently, CCOFs **30** and **31** were determined to possess the architectures presented in Fig. 2. These two CCOFs, derived from the polymerization of BINOL derivative molecules with TMDA and TSDA through imine bonding, exhibit a diamond network with open channels (Fig. 2a and b). Both CCOFs **30** and **31** feature open channels of ∼21.5 × 21.5 Å² and ∼16.6 × 16.6 Å² along the *c*-axis, and ∼2.5 × 11.7 Å² along the *b*-axis (Fig. S6a and b). The two naphthalene rings of the **BDFP-CR6** monomer are twisted along the pivotal C–C bond at the 1,1'-positions with a dihedral angle of 91.7° for **30** and 93.9° for **31** (Fig. S6c and d).

The porosity analysis of CCOFs was conducted by measuring N_2_ sorption isotherms at 77 K on activated samples (Fig. 1c and d). Both CCOFs exhibited a rapid uptake at low pressures (*P*/*P*_0_ < 0.05), signifying their microporous nature. The Brunauer-Emmett-Teller (BET) surface areas were calculated as 325 and 257 m^2^ g^−1^ for CCOFs **30** and **31**, respectively, and the total pore volumes were determined as 0.264 cm^3^ g^−1^ and 0.305 cm^3^ g^−1^. Additionally, the non-local density functional theory (NLDFT) analysis yielded pore widths of around 1.2–2.0 nm for both CCOFs (the inset of Fig. 1c and d).

**Figure 3.**
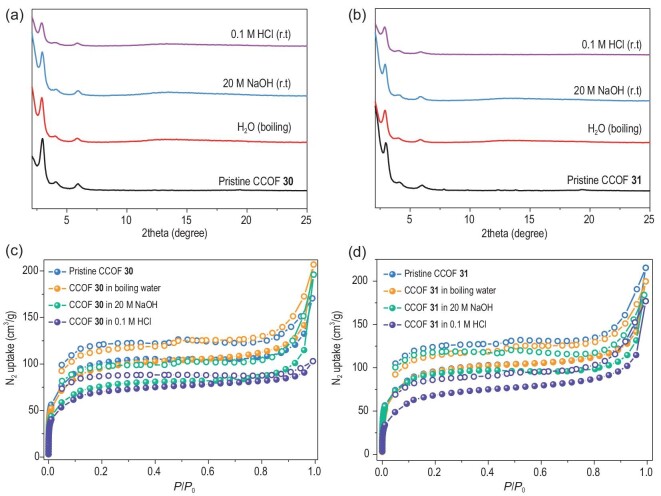
PXRD patterns and N_2_ adsorption-desorption isotherms of (a, c) **30** and (b, d) **31** after treatment in different solvents for 5 days.

Thermogravimetric analysis (TGA) revealed that the two COFs possess good thermal stability, with **30** and **31** remaining stable up to 360°C and 355°C, respectively (Fig. S7). Furthermore, both materials possess excellent chemical stability, as evidenced by unchanged PXRD after immersion in various solutions, including boiling water, 0.1 M HCl and 20 M NaOH aqueous solutions (Fig. 3a and b). Additionally, the CCOF samples treated with water, base and acid exhibited a decrease in BET surface areas of 4%, 23% and 30% for **30**, and 6%, 10% and 20% for **31**, respectively (Fig. 3c and d, and Fig. S8). This further confirms their chemical stability, which is advantageous for their application as CSPs in acidic or basic environments.

### Chiral separation via CEC

The capillary columns coated with CCOFs **30** and **31** were prepared through a previously reported dynamic coating technique (see details in the Supplementary Data) [48]. Before coating the columns, the two CCOFs were manually ground and then dispersed in ethanol to prepare a COF suspension, and the chemical stability of COFs in ethanol was assessed by PXRD and N_2_ adsorption (Figs S9 and S10). The SEM images, as shown in Fig. S11, displayed a consistent distribution of CCOF **30** or **31** on the inner surface of capillary columns, revealing a COF coating thickness ranging from 1.5 to 1.8 μm. In CEC separation, the working voltage is usually up to several thousand or even more than 20 000 volts. Based on this, the impact of operating voltage on the column current needs to be evaluated before conducting the separation experiment. Both the **30**- and **31**-coated columns showed a linear relationship between operating voltage (ranging from +8 to +20 kV) and the generated current (Fig. S12). Thus, the separation experiment can be conducted within the above-mentioned voltage range. Electro-osmotic flow (EOF), as a driving force in CEC, is another indispensable factor. As the pH changed from 3.5 to 8.5, the EOF values for the two CCOF-coated columns and the bare column showed an increasing trend (Fig. S13). Compared to the bare capillary, the EOFs of the two coated capillary columns were slightly smaller. This can be attributed to the fact that the degree of dissociation of silicon hydroxyl groups on the internal wall of the empty capillary is greater than the two CCOF-coated columns. The results also indirectly suggested that CCOFs **30** and **31** were coated on the inner wall of the capillary columns.

**Figure 4.**
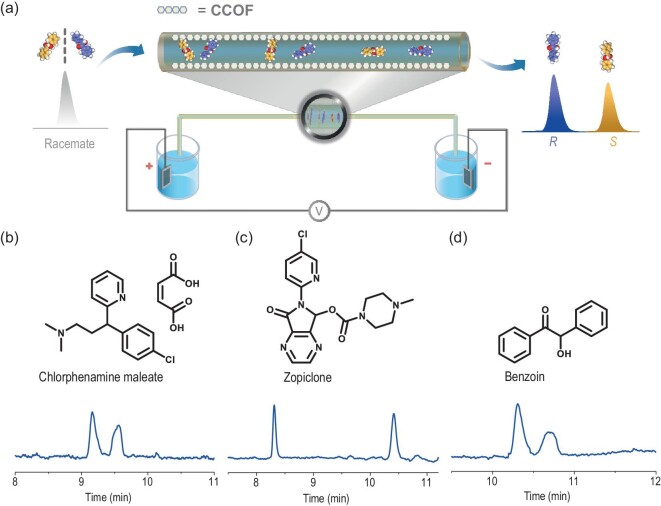
(a) Schematic illustration of CEC separation by CCOF-coated chiral column. (b–d) Representative separation chromatograms of the CCOF **30-**coated open tubular columns for the separation of racemates in CEC.

To explore the chiral separation capability of both columns, chiral compounds were utilized to estimate the performance of enantioseparation (Fig. 4a). Representative separation chromatograms are presented in Fig. 4b–d and Fig. S14. The optimal chromatographic separation conditions and evaluation parameters such as column efficiency (*N*), separation factors (*α*) and the optimal resolution (*Rs*) are exhibited in Table 1. For the CCOF **30**-coated column, three racemates including chlorphenamine maleate, zopiclone and benzoin were well-separated. Specifically, chlorphenamine maleate (∼13.8 × 10.4 Å, Fig. S15) is a medication to prevent allergic diseases (such as rhinitis and urticaria) and can be used as an alkylamine antihistamine. It was separated on this capillary column with *α*/*Rs* = 1.04/2.00. A drug for insomniacs (especially suitable for patients who cannot tolerate residual effects the next morning) called zopiclone (∼14.2 × 8.0 Å) achieved separation by this column with *α*/*Rs* = 1.22/16.55. Benzoin (∼11.9 × 6.3 Å) is an anti-inflammatory and analgesic drug that protects skin wounds from irritation and infection. It could be efficiently separated by a CCOF **30** column with *α*/*Rs* = 1.04/1.60. For the **31**-coated column, three racemates including Tröger's base, zopiclone and *trans*-stilbene oxide were well-separated. Namely, the Tröger's base (∼12.9 × 8.4 Å), *α*/*Rs* = 1.04/2.35 was acquired. Zopiclone, as a medication for short-term insomnia treatment, can be separated (*α* was 1.27 and *Rs* was 20.90). For *trans*-stilbene oxide (∼12.9 × 6.8 Å), *α*/*Rs* = 1.04/1.72. The above-mentioned results demonstrate the good enantioseparation performance of both columns in CEC.

**Table 1.** Optimal CEC separation conditions and chromatographic evaluation parameters of the CCOF **30** and CCOF **31** columns.

		Separation conditions^a^						
	Racemates	U (kV)	pH	*c* (mM)	*N*_1_ (m^−1^)	*N*_2_ (m^−1^)	*α*	*Rs*
CCOF **30**	Chlorphenamine maleate	15	7.5	150	101 600	50 251	1.04	2.00
	Zopiclone	15	6.5	150	204 364	142 535	1.22	16.55
	Benzoin	15	7.5	100	117 459	37 528	1.04	1.60
CCOF **31**	Tröger's base	15	7.5	100	87 710	95 523	1.04	2.35
	Zopiclone	15	6.5	150	234 625	198 297	1.27	20.90
	*Trans*-stilbene oxide	15	7.5	100	86 733	47 774	1.04	1.72

a U is separation voltage; pH is buffer solution pH; *c* is buffer concentration.

Based on the above-mentioned results, there were three chiral drugs (chlorphenamine maleate, zopiclone and benzoin) that were efficiently separated, and an electrochromatographic separation method has been successfully established based on the two capillary columns. They have all been completely separated, especially zopiclone, with an *Rs* of up to 16.5. The chirality of medications and drugs is of great importance in pharmacological and medical research. The potential for practical application of the CCOF **30-** and **31-**coated capillary columns was proven by their successful employment in the enantioseparation processes of commercialized medications, and the feasibility of separated chiral drugs employing CCOF as CSP in CEC was practical. The separation results indicate that they show good separation performance on ketones, epoxides and alkaline substances.

Typically, COFs used as CSPs in chromatography show limited chemical stability, with restricted substrate scope and separation efficiency [19–22,40–43]. For example, when a chiral DA-TD COF (DA is 2,5-dimethoxyterephthalaldehyde; TD is 1,3,5-tri(4-aminophenyl)-2-(2-methylbutoxy)benzene) was used as a CSP in CEC, only three targeted substrates achieved baseline separation, with the highest resolution (*Rs*) being 4.13 [20]. Moreover, there are no reports on the enantioseparation of challenging chiral molecules using COFs as CSPs in CEC [19–22]. In contrast, in our work, all analytes including three drugs achieved baseline separation, with the highest resolution (*Rs*) reaching 16.5.

EOF, as a driving force, plays a crucial role in the transmission of analytes in CEC separation. Many factors (such as separation voltage, buffer concentration and buffer pH) can affect it and then impact the analysis speed of racemates and electrochromatographic separation performance. Herein, the effect of factors that affect the separation performance of racemates, including the applied voltage, concentration of buffer solution, and the buffer pH, was investigated to achieve optimal separation capability.

**Figure 5.**
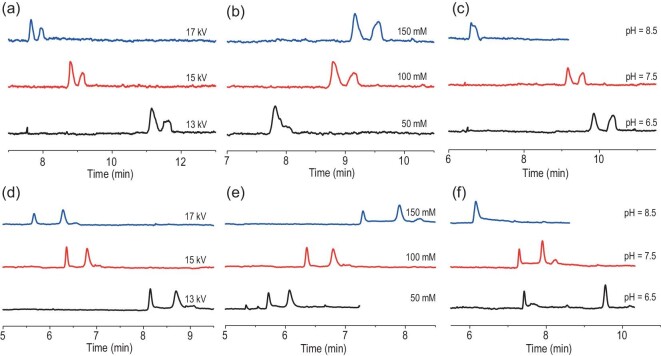
Effects of (a) separation voltage, (b) buffer solution concentration and (c) buffer solution pH on enantioseparation of chlorphenamine maleate racemate in the CCOF **30**-coated capillary column. Effects of (d) applied voltage, (e) buffer solution concentration and (f) buffer pH on enantioseparation of zopiclone racemate in the CCOF **31**-coated capillary column.

The migration rate and retention time of molecules in the mobile phase and the resolution of molecules would be impacted by the separation voltage in CEC separation. To acquire the optimal separation voltage value, we explored the effect of applied voltage on enantioseparation ability of the **30-** and **31**-coated columns. For the **30**-coated column, the *Rs* of chlorphenamine maleate, zopiclone and benzoin was investigated under different separation voltages in Tris-H_3_PO_4_ buffer solution (100 mM and pH = 7.5) (Fig. 5a and Fig. S16). When the applied voltage was changed from 13 to 17 kV with a gradient of 2 kV, the *Rs* of chlorphenamine maleate varied from 1.41 to 1.58 and then to 1.53, the *Rs* of zopiclone increased from 2.74 to 3.50 and then declined to 2.38, and the *Rs* of benzoin changed from 1.42 to 1.60 and then to 1.28, respectively. For the **31-**coated column, the resolutions of Tröger's base, zopiclone and *trans*-stilbene oxide were also investigated under different voltage values in Tris-H_3_PO_4_ buffer solution (100 mM and pH = 7.5) (Fig. 5d and Fig. S16). When the offered voltage value was 13, 15 and 17 kV, the acquired *Rs* of Tröger’s base was augmented from 2.08 to 2.35 and then diminished to 1.45, the *Rs* of zopiclone increased from 3.60 to 4.30 and then shrunk to 3.75, and the obtained *Rs* of *trans*-stilbene oxide changed from 1.52 to 1.72 and then slightly declined to 1.43.

The aforementioned results, as well as Fig. S16 and Table S1, show that the retention times of the above analytes were shortened because of the accelerated migration rate of the mobile phase as the voltage value increased. This change was brought about by the shorter interaction time between the analytes and COF coating due to the increased migration rate of the mobile phase. A non-linear relationship exists between the *Rs* of the analyte and the applied voltage value. Therefore, choosing an appropriate operating voltage value is more conducive to achieving enantiomeric separation in CEC. Taking into account the separation efficiency, including resolution and retention time, etc., the optimal separation voltage was determined to be 15 kV to conduct the subsequent experiment.

Generally, a higher concentration of buffer solution with larger ionic strength can generate smaller EOF. To determine proper concentration value, the effect of buffer concentration on separation performance was explored with the two COF-coated capillaries. For the **30**-coated column, chlorphenamine maleate, zopiclone and benzoin were separated in Tris-H_3_PO_4_ buffer solutions (50, 100 and 150 mM) with a pH of 7.5 under 15 kV voltage (Fig. 5b and Fig. S17). Practically speaking, when the buffer concentration was 50 mM, only zopiclone was well separated (*Rs* = 2.19), while the other two analytes failed to separate. With the concentration increased to 100 mM, the resolution of zopiclone rose to 3.50. Meanwhile, chlorphenamine maleate and benzoin achieved separation. The *Rs* were 1.58 and 1.60, respectively. When the concentration was changed to 150 mM, the separation performance of chlorphenamine maleate (*Rs* was 2.00) and zopiclone (*Rs* was 5.38) was better than those for 100 mM of buffer solution, while the resolution of benzoin was slightly declined (*Rs* = 1.55).

For the **31**-coated column, the resolutions of Tröger's base, zopiclone and *trans*-stilbene oxide were also studied in Tris-H_3_PO_4_ buffer solution (50, 100 and 150 mM) with a pH of 7.5, under 15 kV voltage (Fig. 5e and Fig. S17). Briefly, when the buffer solution concentration was 50 mM, the *Rs* of Tröger's base was 1.09, zopiclone was 3.62 and *trans*-stilbene oxide was 1.02. When the concentration was increased to 100 mM, the obtained *Rs* of the three analytes was 2.35, 4.30 and 1.72, respectively, and was augmented. As the concentration was raised from 100 to 150 mM, Tröger's base and *trans*-stilbene oxide acquired diminished resolutions (*Rs* was 1.45 and 1.22, respectively), while the zopiclone obtained an increased resolution (*Rs* was 5.10). Considering the trend of changes in resolution and retention time of analytes at different buffer concentrations, the optimal buffer solution concentration was 100 mM for the separation of benzoin, Tröger's base and *trans*-stilbene oxide.

As the concentration of buffer solution changed from 50 to 150 mM, there was an increased tendency towards the separation performance of chlorphenamine maleate and zopiclone. Based on this, the buffer concentration was further increased to enhance separation capability. However, as the concentration reached 175 mM, the separation voltage was extremely unstable and the experiment was halted. Generally, a higher concentration buffer solution possesses a higher ionic strength, and the electrical double layer formed on the inner surface of the column is thinner, with a smaller Zeta potential, resulting in a smaller EOF which is beneficial for CEC separation. However, the thermal effects generated by high buffer concentration cause the separation voltage to be extremely unstable, which is detrimental to the separation experiment. As the concentration was increased within limits, the retention times and resolutions of the analytes were increased (Table S2). At inappropriate buffer concentrations, the racemate cannot achieve optimal separation, and may even fail to separate. Hence, the optimal concentration of the enantioseparation of chlorphenamine maleate and zopiclone was determined to be 150 mM.

Controlling the pH of the buffer system will change the value of EOF, thereby affecting the CEC separation performance. To investigate this, the pH value was studied as a variable in the chromatographic conditions. For the CCOF-**30**-coated column, the resolutions of chlorphenamine maleate, zopiclone and benzoin were investigated under 15 kV voltage in Tris-H_3_PO_4_ buffer solution (100 or 150 mM) with different acidities (Fig. 5c and Fig. S18). Briefly, when the pH of the buffer solution was 6.5, the resolution of the above three racemates was 1.89, 16.55 and 1.02, respectively. When the pH increased to 7.5, the resolution of chlorphenamine maleate and benzoin increased to 2.00 and 1.60, respectively, while the zopiclone sharply decreased to 5.38. When the pH changed to 8.5, the chlorphenamine maleate, zopiclone and benzoin failed to separate. Given that the resolution of zopiclone showed a declined trend when the pH rose from 6.5 to 8.5, we tested the resolution of zopiclone at a slightly lower pH 5.5 to achieve a better separation performance. Unfortunately, the resolution of zopiclone still decreased to 3.46.

For the **31**-coated column, Tröger's base, zopiclone and *trans*-stilbene oxide were also separated under 15 kV in 100 or 150 mM Tris-H_3_PO_4_ buffer solution with different acidities (Fig. 5f and Fig. S18). Concretely, at pH 6.5, the resolutions of the above three racemates listed in sequence were 1.47, 20.90 and 1.61, respectively. When the pH was 7.5, the resolutions of Tröger's base and trans-stilbene oxide increased to 2.35 and 1.72, respectively, while the zopiclone sharply decreased to 5.10. When the pH further changed to 8.5, the Tröger's base, zopiclone and *trans*-stilbene oxide failed to separate. For the zopiclone, its resolution displayed a large decrement when the pH increased from 6.5 to 8.5. So, we separated the zopiclone in 150 mM Tris-H_3_PO_4_ buffer solution at a slightly lower pH of 5.5 to gain better separation performance. However, the resolution of zopiclone still showed a tendency to decline, and decreased from 20.90 to 16.21. Therefore, the optimal separation pH of chlorphenamine maleate, benzoin, Tröger's base and *trans*-stilbene oxide was 7.5, and zopiclone was 6.5, respectively. Generally, the dissociation degree of silicon hydroxyl groups increased with the increase of pH between 4.0 and 10.0, and the EOF was also enhanced [49]. The migration times of analytes utilized in the experiment showed a reduced trend as the buffer pH increased (Fig. S18 and Table S3). Poor resolution was frequently attained because of the reduced contact time between the analytes and the COF coating caused by the increased EOF. So, an appropriate pH value is necessary.

Additionally, the pH of the buffer can alter the charge of the analyte and the COF coating. The impact of pH values between 6.5 and 8.5 was studied (Fig. S18). Zopiclone and benzoin were less positively charged with the pH increase, but their retention time was decreased, which may be due to the changed ionization levels of the hydroxyl group and tertiary amine, and due to an increase of EOF. Generally, the pKa values for the hydroxyl group and tertiary amine were between 8.0 and 12.0, and 9.0 and 11.0, respectively. When the buffer pH was below pKa, the hydroxyl group ionization degree increased as the pH changed from 6.5 to 8.5, whereas tertiary amine protonation level reduced. The strength of the interaction between the analytes with the CCOF coating weakened. Meanwhile, the other interaction forces (including dispersion forces, hydrophobic effect and *π*-*π* interactions) between the analytes and CCOF coating may also play a role in the capillary electrochromatographic separation. Therefore, offering an appropriate buffer pH is vital in CEC separation. From the abovementioned optimization of enantioseparation conditions, we can see that the applied voltage, buffer concentration and acidity of buffer solution were vital factors that can affect the separation performance of racemates in CEC. To evaluate the repeatability of CCOF **30-** and **31**-coated capillaries, chlorphenamine maleate and Tröger's base were used as the analytes, respectively (Fig. S19). Relative standard deviations (RSD) of retention time and resolution of chlorphenamine maleate were 5.2% and 16.2%, and those of Tröger's base were 7.3% and 13.1%, respectively.

**Figure 6.**
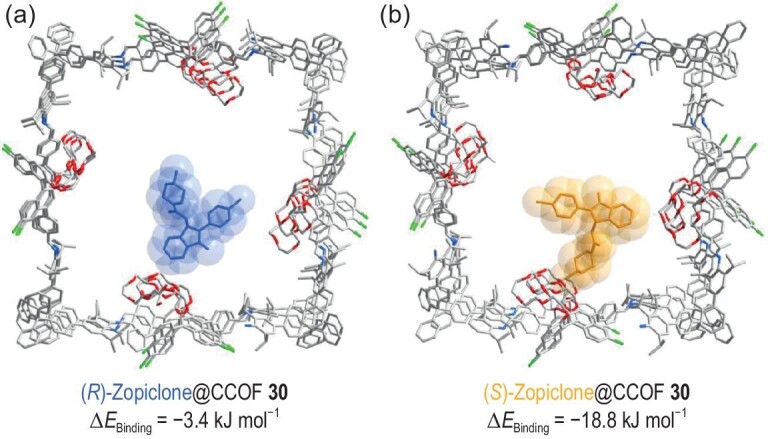
(a, b) Top views of host-guest conformations of (*R*)-zopiclone@CCOF **30** and (*S*)-zopiclone@CCOF **30** based on energy-minimized DFT calculations. The relative binding energies are indicated.

In order to microscopically elucidate the host–guest interactions between the CCOF and enantiomers to reveal the chiral separation mechanism, we tentatively studied COF–enantiomer interactions through energy-minimized DFT calculations. CCOF **30** was utilized as the host model, and the binding energies of (*R*)-/(*S*)-zopiclone were evaluated by inserting a single zopiclone molecule into the CCOF channels and conducting energy minimization for the zopiclone@**30** structures. The optimized structures showed that both (*R*)- and (*S*)-zopiclone are combined with the open cavity of crown ether groups, primarily governed by multiple CH-*π* interaction forces and hydrophobic interaction forces between the crown ether groups of **30** (host) and zopiclone (guest) (Fig. 6). The binding energies of the host–guest structures, (*R*)-zopiclone@**30** and (*S*)-zopiclone@**30**, were calculated to be −3.4 and −18.8 kJ mol^−1^, respectively. The negative values of the binding energies demonstrated that the COF nanochannel containing crown ether groups is favorable for the combination of guest molecules. The more negative binding energy for (*S*)-zopiclone compared to (*R*)-zopiclone suggests a stronger host–guest binding of CCOF **30** with (*S*)-zopiclone, leading to efficient chromatographic separation. These theoretical calculations are in good agreement with the experimental results of CEC enantioseparation.

## CONCLUSIONS

In summary, we showcased the design of two crown-ether-decorated 3D CCOFs by constructing isopropyl-protected imine linkages between tetrahedral tetraamines and an enantiopure 1,1'-binaphyl-20-crown-6-derived dialdehyde. Comprehensive characterization techniques, including PXRD, N_2_ adsorption, ^13^C NMR, SEM, TEM and molecular modeling, confirmed the structural integrity and unique features of the CCOFs. These frameworks exhibit interpenetrated open structures with tubular channels decorated with chiral crown ether groups. Both CCOFs functioned effectively as CSPs, showing exceptional selectivity in CEC-based separation of challenging enantiomers, particularly racemic drugs. The incorporation of rich crown ether recognition sites and the confinement effect of robust porous COF structures contributed to their outstanding selectivity and stability in resolving racemates. This study combines the advantages of COFs and crown ethers, putting COFs forward as highly stable platforms for new CSP design, offering great promise for practical electrochromatographic enantioseparation.

## Supplementary Material

nwae256_Supplemental_Files
