# Supplementary material for: Construction of chiral crown ethers in robust covalent organic frameworks for electrochromatographic enantioseparation
Source: Natl Sci Rev. 2024 Jul 26;11(10):nwae256. doi: 10.1093/nsr/nwae256 (PMC11879391; doi:10.1093/nsr/nwae256)
Supplement: nwae256_Supplemental_Files [file nwae256_supplemental_files.zip › SI-MS-2024-557-R3.pdf]

Supporting Information for

Construction of chiral crown ethers into robust covalent organic frameworks for electrochromatographic enantioseparation

Shiguo Fu,[a,†] Gaizhao Qin,[b,†] Jinqiao Dong,[a] Chen Yuan,[a] Yan Liu,[a] Li-Ming Yuan,[b] and Yong Cui[a,*]

[a]*School of Chemistry and Chemical Engineering and State Key Laboratory of Metal Matrix Composites, Shanghai Jiao Tong University, Shanghai 200240, China*

[b]*Department of Chemistry, Yunnan Normal University, Kunming 650500, P. R. China*

[†]S. Fu and G. Qin contributed equally to this paper.

Email: yongcui@sjtu.edu.cn

Table of Content

1. Materials and general procedures
2. Synthesis
3. Figure S1. FT-IR spectra
4. Figure S2. Solid-state ^{13}C NMR spectra
5. Figure S3. The CD spectra
6. Figure S4. Structures modeling
7. Figure S5. TEM images
8. Figure S6. Additional structural figures.
9. Figure S7. TGA curves
10. Figure S8. BET plots.
11. Figure S9-10. Open-tubular capillary column packing.
12. CEC procedures.
13. Figure S11-13. Characteristic of the capillary columns.
14. Calculation of separation factor and resolution.
15. Figure S14. The CEC chromatograms.
16. Figure S15. The space-filling models of racemates.
17. Figure S16-18. The effects of separation conditions.
18. Repeatability. Figure S19.
19. Fractional atomic coordinates and unit cell parameters.
20. References.

1. Materials and general procedures.

All the chemicals are commercially available, and used without further purification. All solvents were dried and distilled according to standard laboratory methods. Powder X-ray diffraction data (PXRD) were collected on a Bruker D8 Advance diffractometer using Cu Kα radiation. ^{1}H and ^{13}C NMR were measured using a MERCURY plus 400 spectrometer operating at resonance frequencies of 400 MHz. Solid ^{13}C NMR experiments were acquired by using an AVANCE III HD 400 Bruker BioSpin Corp. The FT-IR (KBr pellet) spectra were recorded on a Spectrum 100, Perkin Elmer, Inc. Thermogravimetric analyses (TGA) were carried out in an air atmosphere with a heating rate of 10 ^{o}C/min on a Q5000IR, TA Instruments, USA. The CD spectra were measured on the JASCO J-800 spectrophotometer. Scanning Electron Microscopy (SEM) images were recorded on a Nova NanoSEM 450, FEI. High-resolution transmission electron microscopy (HRTEM) images were characterized on a Field Electron and Ion Company (FEI) Talos F200X G2 electron microscope. The porous properties of the covalent organic frameworks (COFs) were investigated by nitrogen adsorption and desorption at 77 K using ASAP 2020, Micromeritics Instrument Corp, USA. The pore-size distribution curves were obtained from the adsorption branches using non-local density functional theory (NLDFT) method. Before the adsorption measurement, the samples were activated at 120 ^{o}C under vacuum (< 10^{-3} torr) for 10 h. Analytical capillary electrochromatography (CEC) experiments were performed on an HPCE system (CL1020, Beijing Huayangliming Instrumental, Ltd., China) equipped with UV detector (190-700 nm). The tested samples were dissolved in the mobile phase solvent (Tris-H_3PO_4 buffer solution) with a concentration of 1 mg/mL. Chromatographic workstation was Hw-2000 (Shanghai qianpu software, China), used for controlling and data collection.

2. Synthesis.

S1, S2, S3, TMDA was synthesized according to the literature.[1-4]

Synthesis of TSDA.

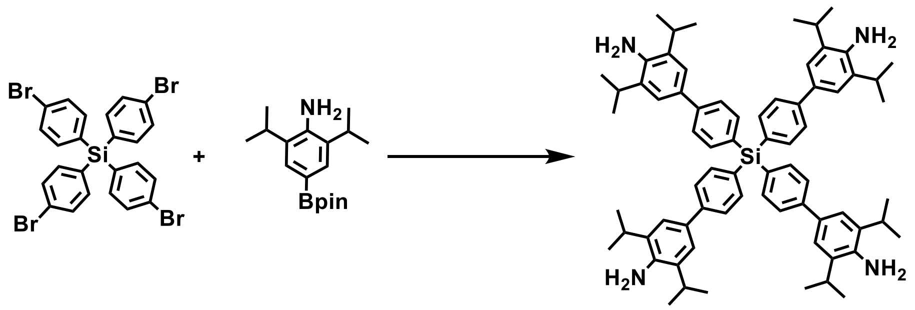

S1 S2 TSDA

1,1',1'',1'''-Silanetetrayltetrakis[4-bromobenzene] **S1** (2 g, 3.06 mmol), 2,6-diisopropyl-4-(4,4,5,5-tetramethyl-1,3,2-dioxaborolan-2-yl)aniline **S2** (4.84 g, 15.97 mmol), KOAc (5.97, 60.87 mmol) and Pd(dppf)Cl_2 (200 mg, 0.245 mmol) in THF/H_2O (3/1, v/v, 120 mL) were degassed for 5 min. The suspension was stirred under N_2 for overnight. The mixture was cooled to room temperature, diluted with water, and extracted with DCM. The organic phase was washed with brine, dried over anhydrous Na_2SO_4 and then concentrated under reduced pressure. The acquired crude product was purified by column chromatography on silica gel (petroleum ether/dichloromethane, 3:1, v/v) to afford **TSDA** as a white solid (1.59 g, 50%). ^{1}H NMR (400 MHz, $CDCl_3$) δ 7.66 (dd, *J* = 7.8, 44.7 Hz, 16H), 7.32 (s, 8H), 3.82 (s, 8H), 3.01-2.94 (m, 8H), 1.33, (d, *J* = 6.8 Hz, 48H). ^{13}C NMR (101 MHz, CDCl3) δ 143.20, 140.05, 136.82, 132.71, 131.94, 131.25, 126.10, 121.90, 28.15, 22.52.

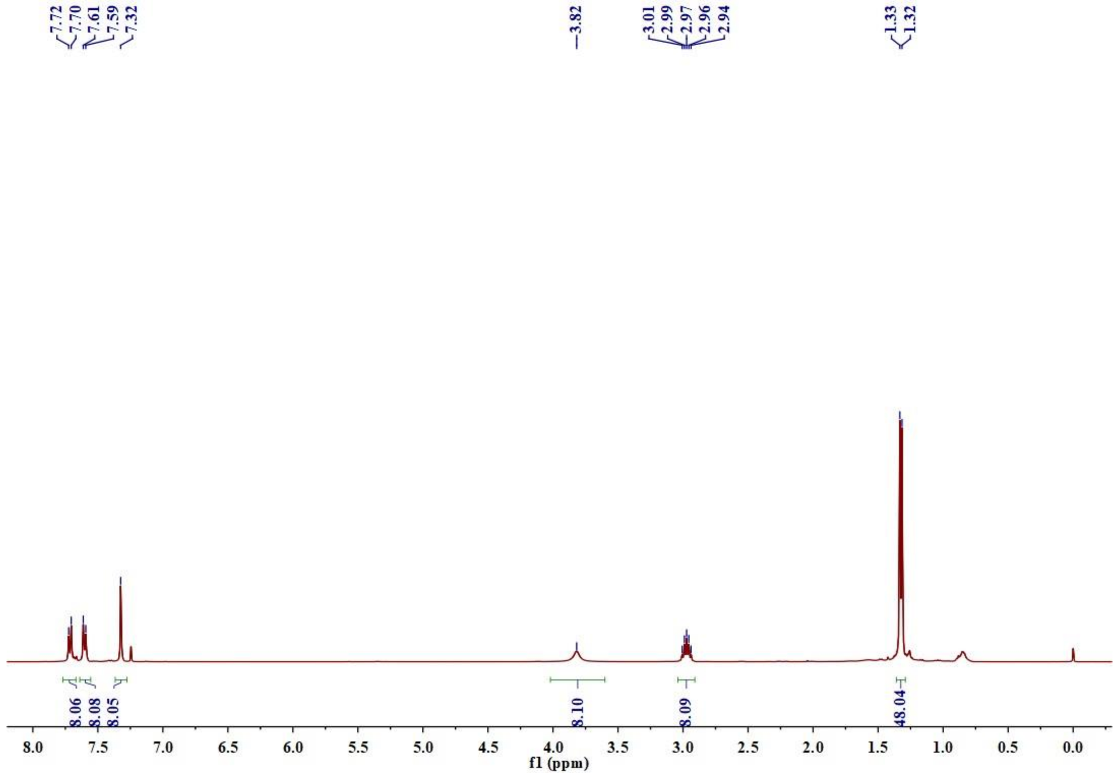

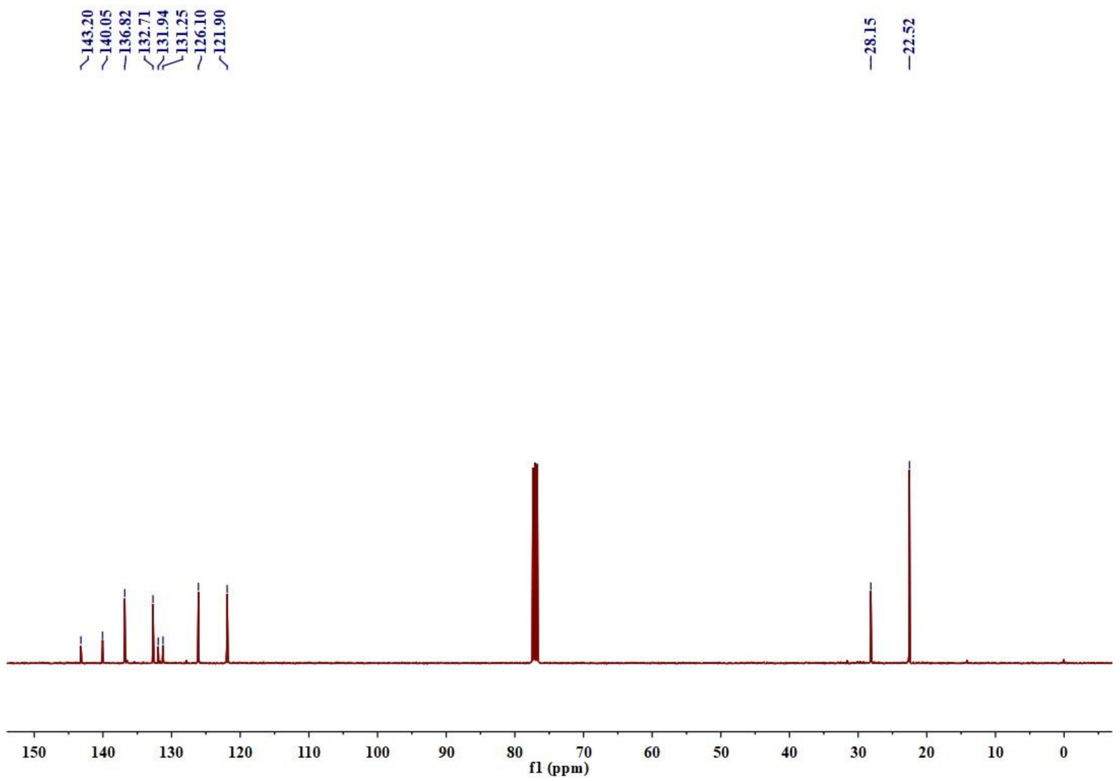

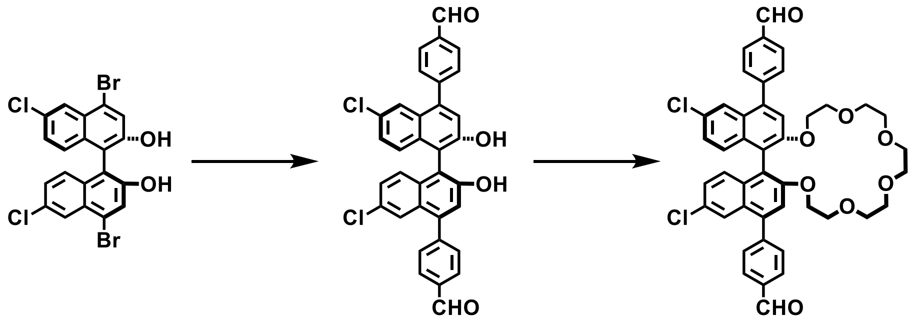

Synthesis of (*S*)-BDFP. (*S*)-4,4'-dibromo-6,6'-dichloro-1,1'-binaphthyl-2,2'-diol **(*S*)-S3** (2.5 g, 4.87 mmol), (4-formylphenyl)boronic acid (2.98 g, 19.8 mmol), K_2CO_3 (4.05 g, 29.3 mmol) and Pd(dppf)Cl_2 (240 mg, 0.294 mmol) in 1,2-dimethoxyethane/H_2O (2/1, v/v, 90 mL) were degassed for 10 min. The suspension was stirred under N_2 at 90 °C for overnight. After cooling to room temperature, the mixture was extracted with DCM. The organic phase was washed with saturated brine, dried over anhydrous Na_2SO_4 and then concentrated under reduced pressure. The obtained crude product was purified by column chromatography on silica gel (petroleum ether/ethyl acetate,

3:1, v/v) to afford **(*S*)-BDFP** as a pale yellow solid (1.97 g, 72%). ^{1}H NMR (400 MHz, DMSO-d_6) δ 10.22 (s, 2H), 9.92 (br, 2H), 8.20 (d, *J* = 8.1 Hz, 4H), 7.90 (d, *J* = 8.0 Hz, 4H), 7.71 (d, *J* = 2.1 Hz, 2H), 7.44 (s, 2H), 7.40 (dd, *J* = 2.2, 9.1 Hz, 2H), 7.20 (d, *J* = 9.1 Hz, 2H). ^{13}C NMR (101 MHz, DMSO-d_6) δ 193.35, 153.48, 145.72, 139.32, 135.98, 133.41, 130.98, 130.39, 128.39, 127.68, 127.34, 126.82, 124.31, 121.01, 115.99. **(*R*)-BDFP** was synthesized following the same method mentioned above except that **(*R*)-S3** was used instead of **(*S*)-S3**.

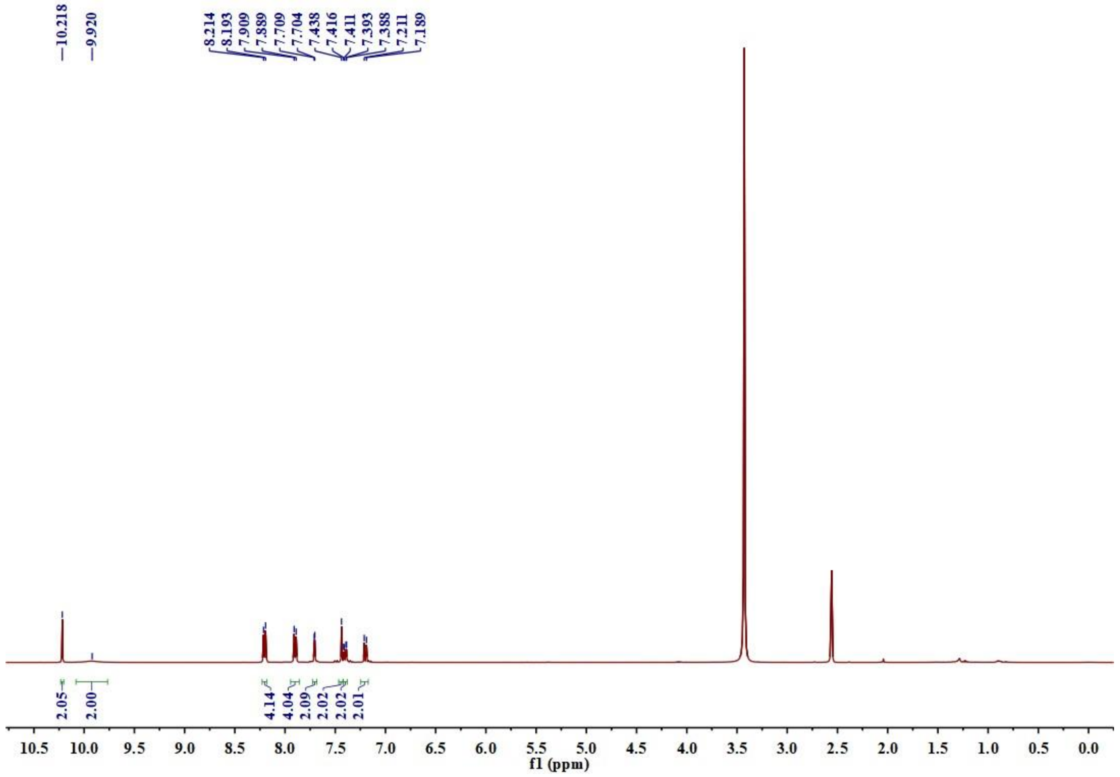

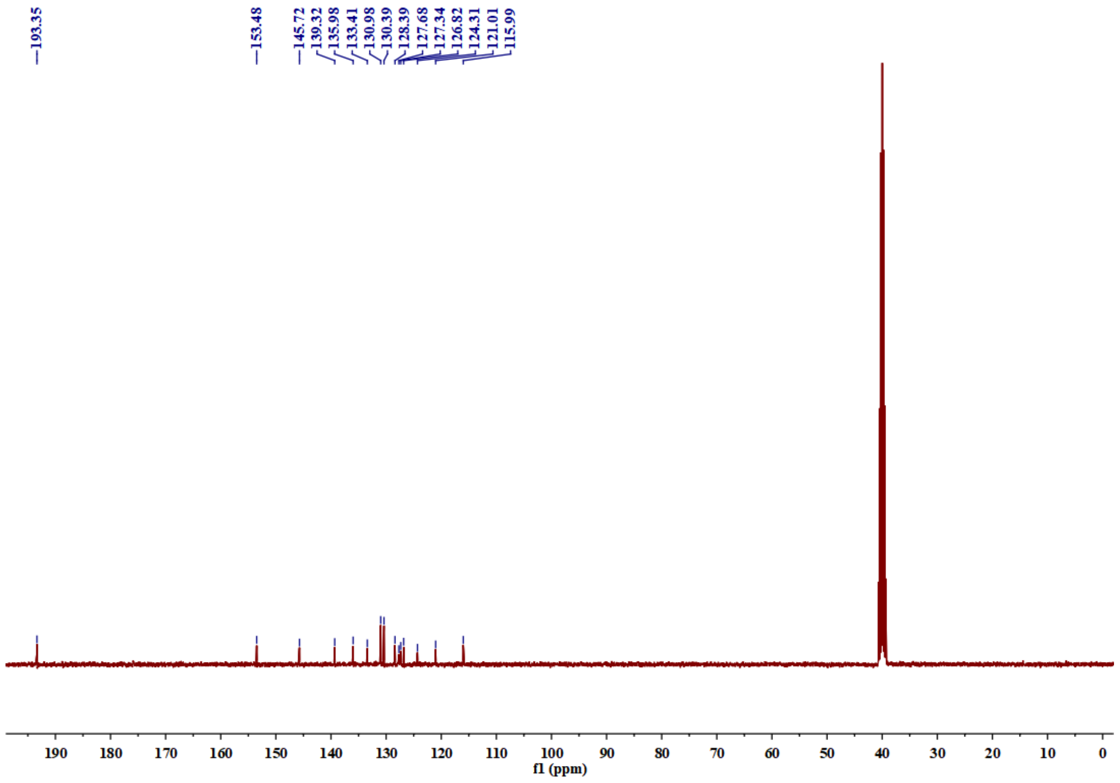

Synthesis of (*S*)-BDFP CR6. **(*S*)-BDFP** (2.00 g, 3.54 mmol), penta(ethylene glycol) di-*p*-toluenesulfonate (2.9 g, 5.30 mmol), Cs_2CO_3 (4.60 g, 14.20 mmol) in DMF (100 mL) were degassed for 10 min. The suspension was stirred under N_2 at 75 °C for 18 h. Cooling it to room temperature, removed DMF, the mixture was extracted with DCM. The organic phase was washed with saturated brine, dried over anhydrous Na_2SO_4 and then concentrated under reduced pressure. The obtained crude product was purified by column chromatography on silica gel (petroleum ether/ethyl acetate, 2:1, v/v) to afford **(*S*)-BDFP CR6** as a white solid. (1.65 g, 61%). ^{1}H NMR (400 MHz, DMSO-d_6) δ 10.18 (s, 2H), 8.17 (d, *J* = 8.0, 4H), 7.92 (d, *J* = 8.0, 4H), 7.75 (d, *J* = 6.1, 4H), 7.39 (d, *J* = 9.1, 2H), 7.16 (d, *J* = 9.1, 2H), 4.36-4.31 (m, 2H), 4.20-4.15 (m, 2H), 3.63-3.58 (m, 2H), 3.51-3.46 (m, 2H), 3.44-3.38 (m, 2H), 3.34-3.26 (m, 10H). ^{13}C NMR (101 MHz, DMSO-d_6) δ 193.38, 154.70, 145.66, 139.73, 136.03, 132.73, 131.29, 130.31, 129.52, 128.00, 127.68, 127.45, 124.37, 119.54, 118.95, 70.56, 70.35, 70.19, 70.01, 69.71. **(*R*)-BDFP CR6** was synthesized following the same method mentioned above except that **(*R*)-BDFP** was used instead of **(*S*)-BDFP**.

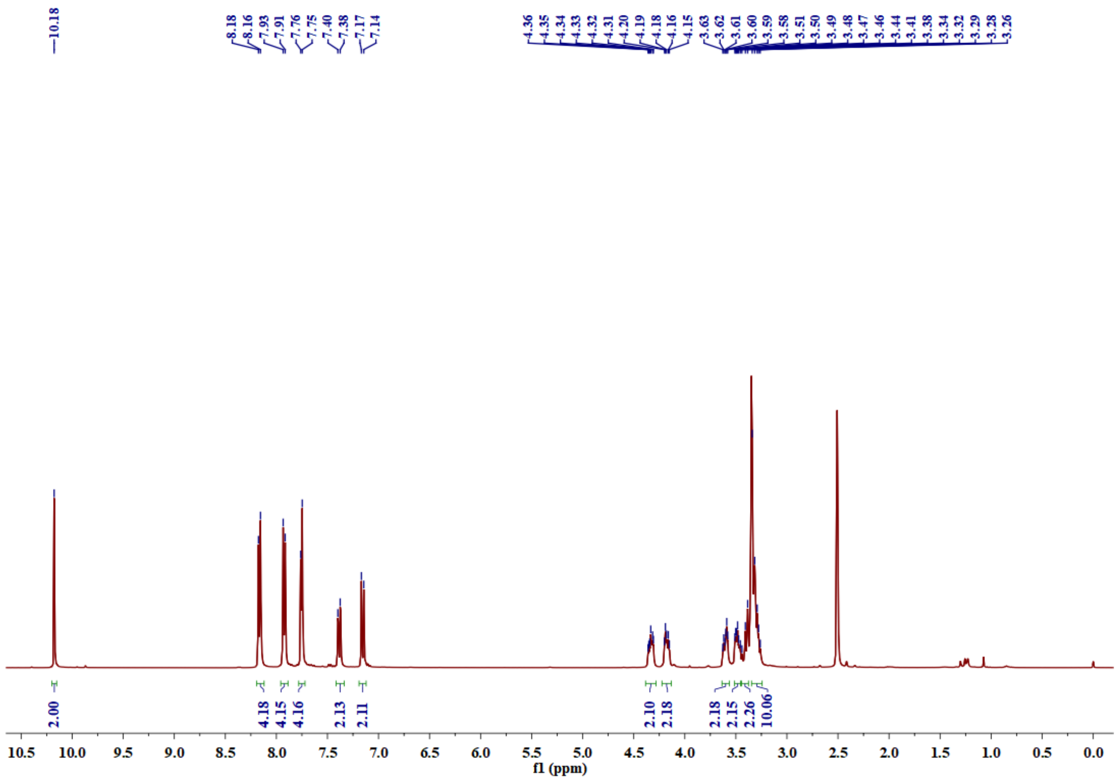

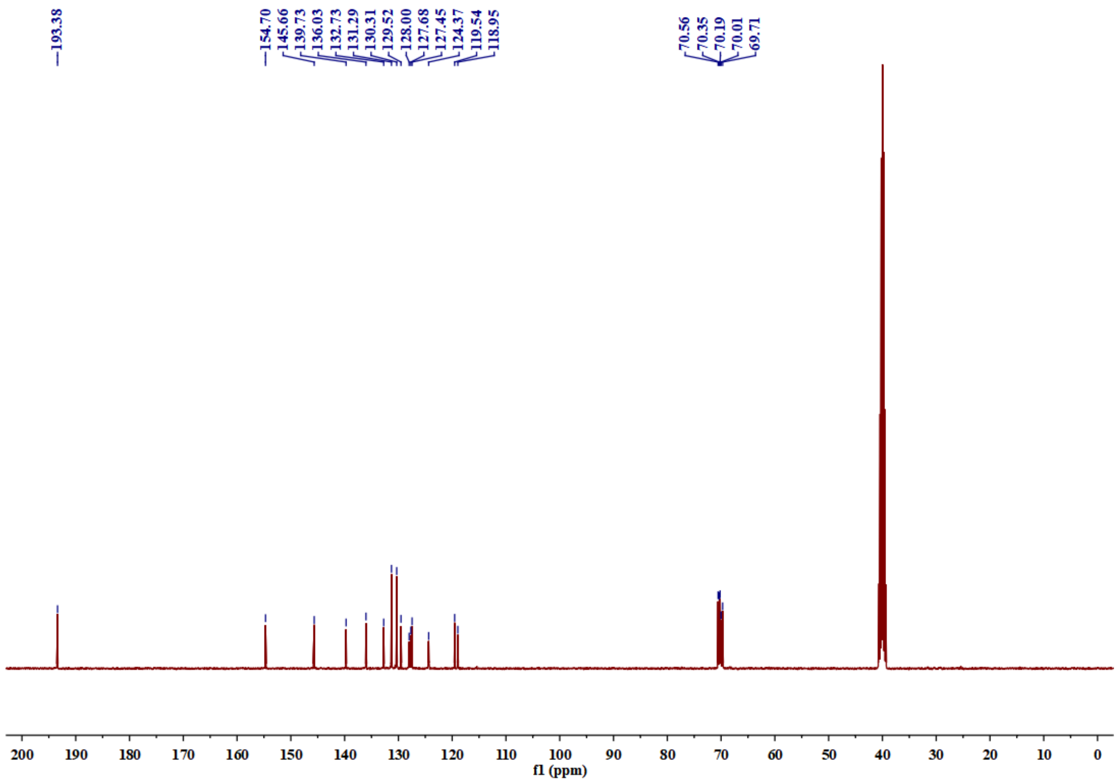

Synthesis of CCOF 30.

A 10 mL Schlenk tube was charged with (*S*)-**BDFP CR6** (17.0 mg, 0.022 mmol), tetraphenyl methane incorporating 2,6-diisopropyl aniline

(4',4''',4''''',4''''''' -methanetetrayltetrakis(3,5-diisopropyl-[1,1'-biphenyl]-4-amine, **TMDA**) (11.5 mg, 0.011 mmol), *iso*-propanol (1.0 mL), mesitylene (0.11 mL) and acetonitrile (0.02 mL). The resulting mixture was sonicated for 10 min. The *p*-toluenesulfonic acid solution (0.10 mL) was then added, and the Schlenk tube was flash frozen at 77 K using the liquid nitrogen bath, evacuated and sealed by Teflon valve. Upon warming to room temperature, the Schlenk tube was heated at 120 ℃ for 3 days. The resulting yellow solid was isolated by centrifugation and washed with DMF (3 × 10 mL), THF (3 × 10 mL) and Et_2O (3 × 10 mL). Further purification of COF was carried out by Soxhlet extraction in THF for 12 h. The powder was dried at 60 ℃ under vacuum overnight to afford CCOF **30** as yellow powder (25 mg, 87%).

Synthesis of CCOF 31

A 10 mL Schlenk tube was charged with (*S*)-**BDFP CR6** (17.0 mg, 0.022 mmol), tetraphenyl silane incorporating 2,6-diisopropyl aniline (4',4''',4''''',4''''''' -silanetetrayltetrakis(3,5-diisopropyl-[1,1'-biphenyl]-4-amine, **TSDA**) (11.5 mg, 0.011 mmol), *iso*-propanol (1.0 mL), *m*-xylene (0.16 mL) and acetonitrile (0.02 mL). The resulting mixture was sonicated for 10 min. *p*-toluenesulfonic acid solution (0.10 mL) was then added, and the Schlenk tube was flash frozen at 77 K using the liquid nitrogen bath, evacuated and sealed by Teflon valve. Upon warming to room temperature, the Schlenk tube was heated at 120 ℃ for 3 days. The resulting yellow solid was isolated by centrifugation and washed with DMF (3 × 10 mL), THF (3 × 10 mL) and Et_2O (3 × 10 mL). Further purification of COF was carried out by Soxhlet extraction in THF for 12 h. The powder was dried at 60 ℃ under vacuum overnight to afford CCOF **31** as yellow powder (23 mg, 81%).

3. Figure S1. FT-IR spectra of CCOFs 30 and CCOF 31.

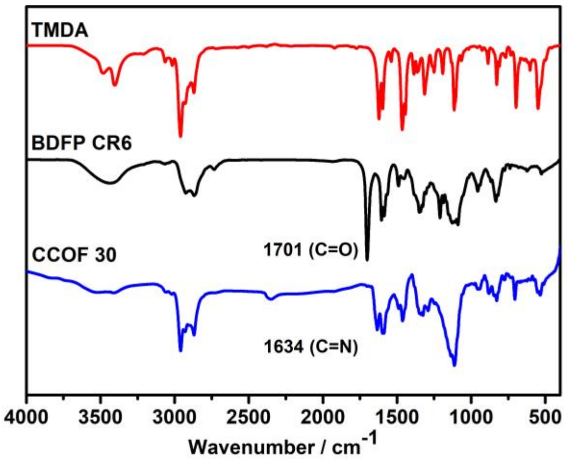

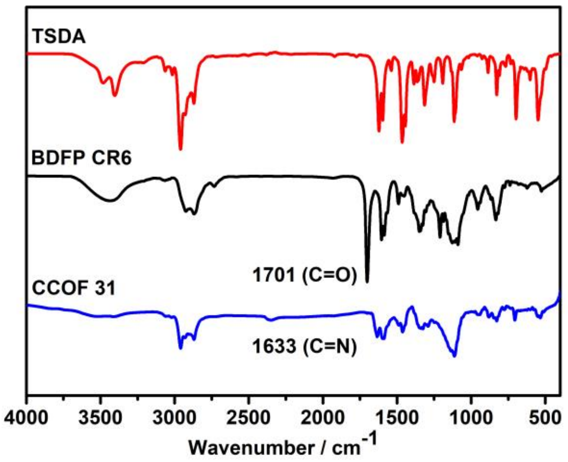

4. Figure S2. Solid-state ^{13}C NMR spectra of CCOFs 30 and CCOF 31.

CCOF 30

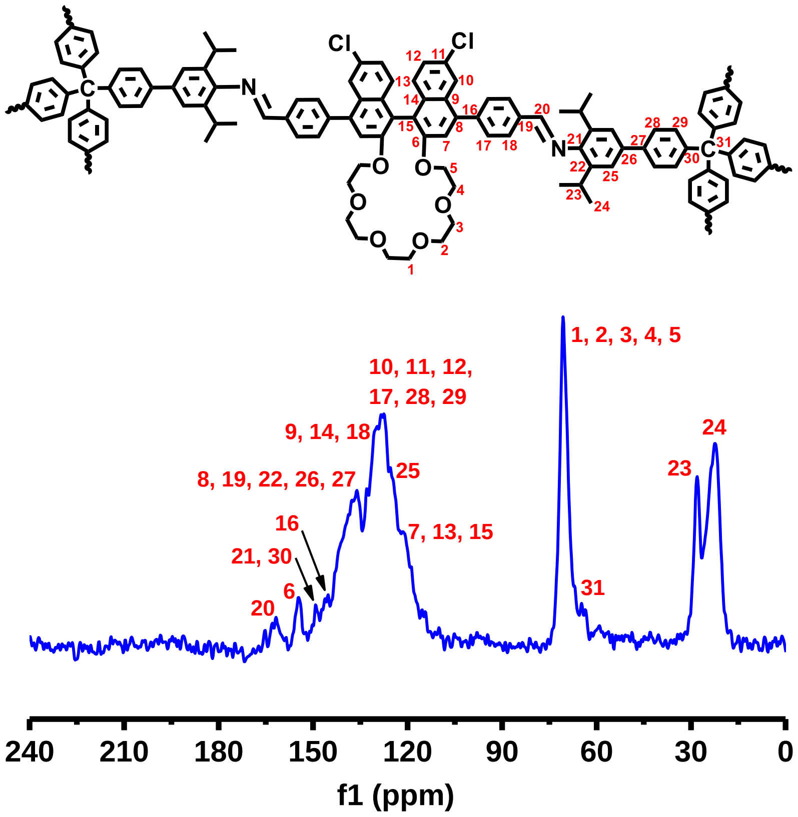

CCOF 31

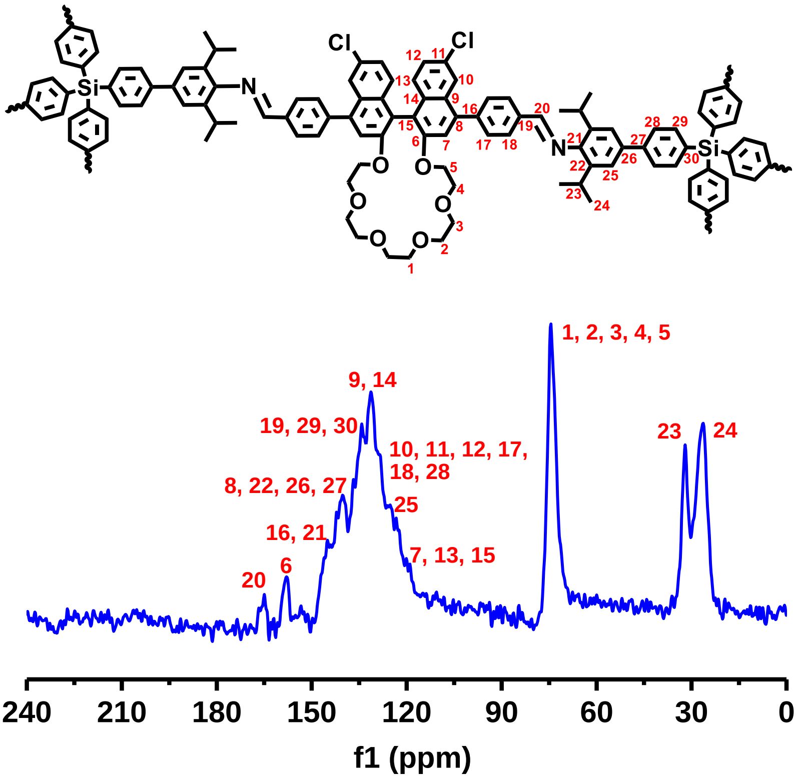

5. Figure S3. The CD spectra of CCOF 30, CCOF 31, and monomer BDFP-CR6.

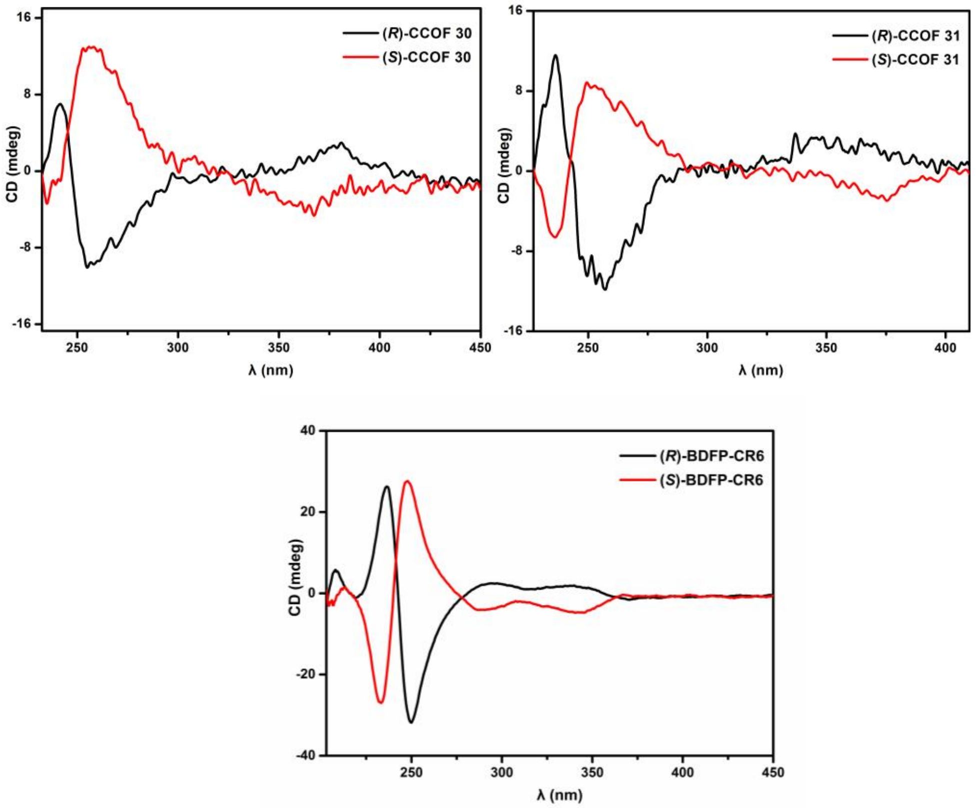

6. Structures modeling

Molecular modeling of these CCOFs was generated with the Materials Studio (version 7.0) suite of programs. Pawley refinement was carried out using Reflex, a software package for crystal determination from PXRD pattern. Unit cell dimension was set to the theoretical parameters. The Pawley refinement was performed to optimize the lattice parameters iteratively until the R_{wp} value converges and the overlay of the observed with refined profiles shows good agreement. The lattice models (cell parameters, atomic positions, and total energy) were then fully optimized using MS Forcite molecular dynamics module (universal force fields, Ewald summations) method.

For this two BINOL-based CCOFs **30** and **31**, considering the geometry of the monomers and the connection patterns of building blocks, we simulated the structures with 4+2 diamond net from zero-fold to 13-fold interpenetration. The calculated PXRD patterns of 11-fold model are in great agreement with the experimental data (**Figure S4**).

Figure S4. Calculated PXRD patterns of (a) CCOF 30 and (b) CCOF 31 based on different interpenetration of a diamond net.

(a)

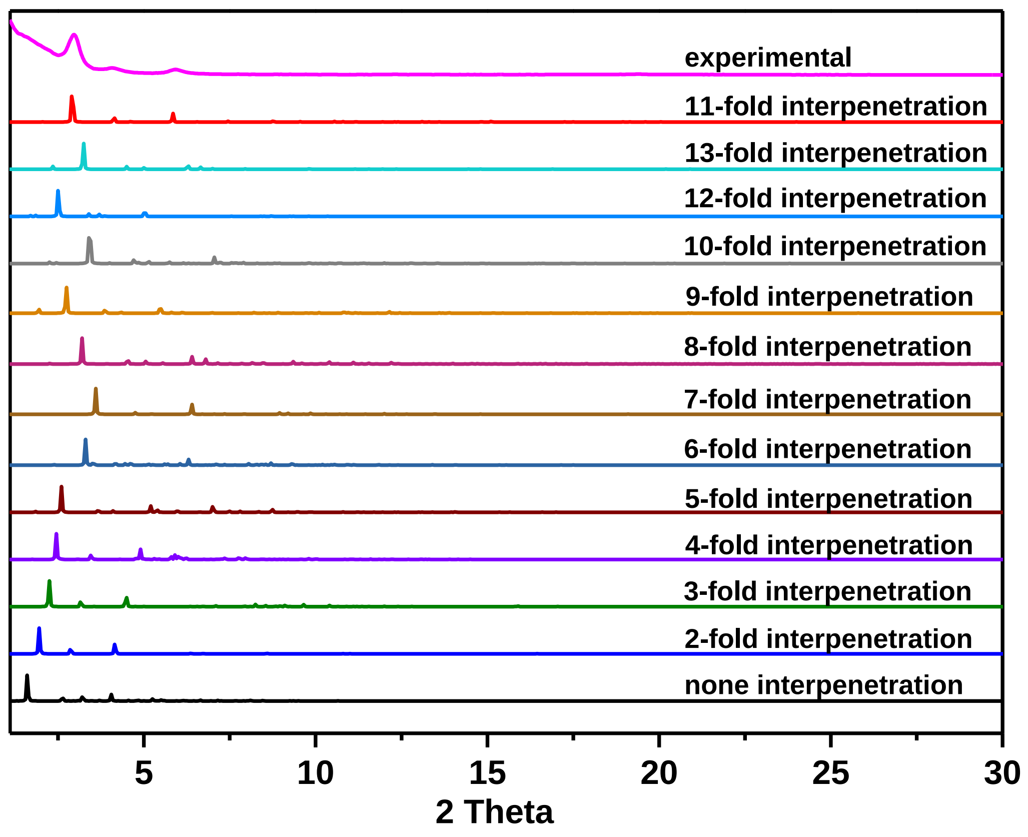

(b)

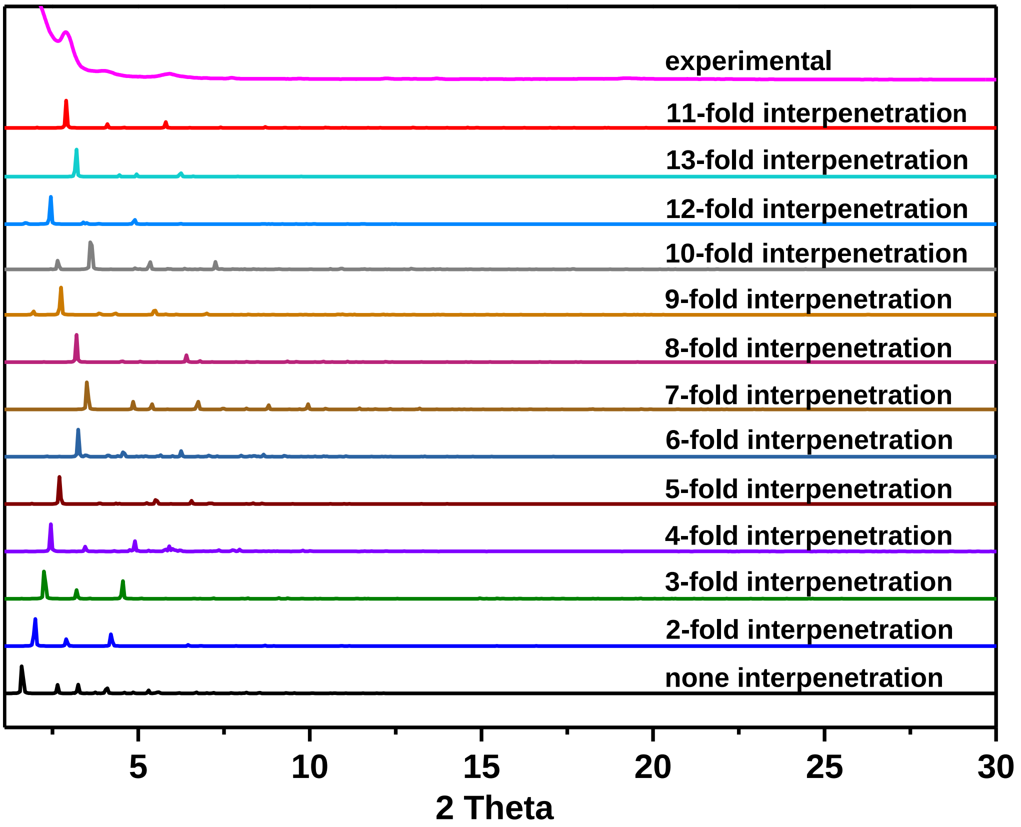

7. Figure S5. TEM images of CCOF 30 and CCOF 31.

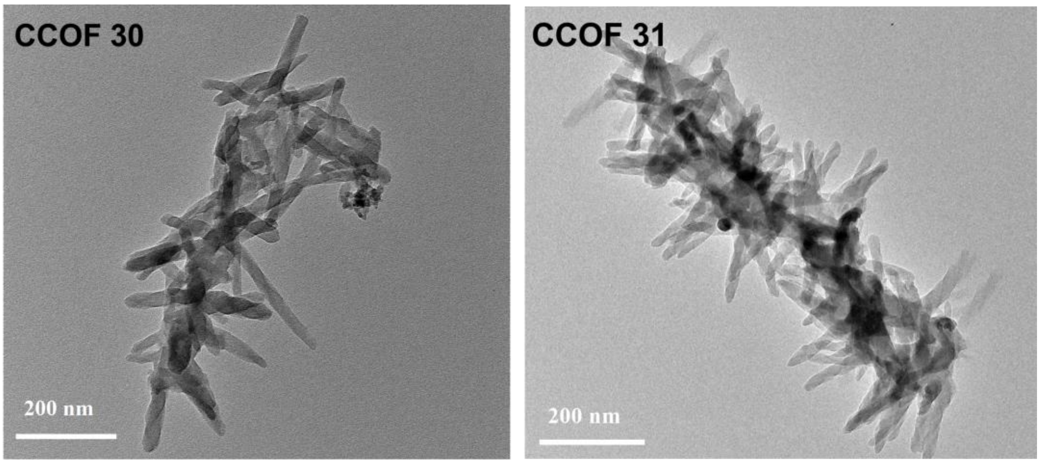

8. Figure S6. Additional structural figures.

(a) The pore size of CCOF **30** along *c*-axis and *b*-axis.

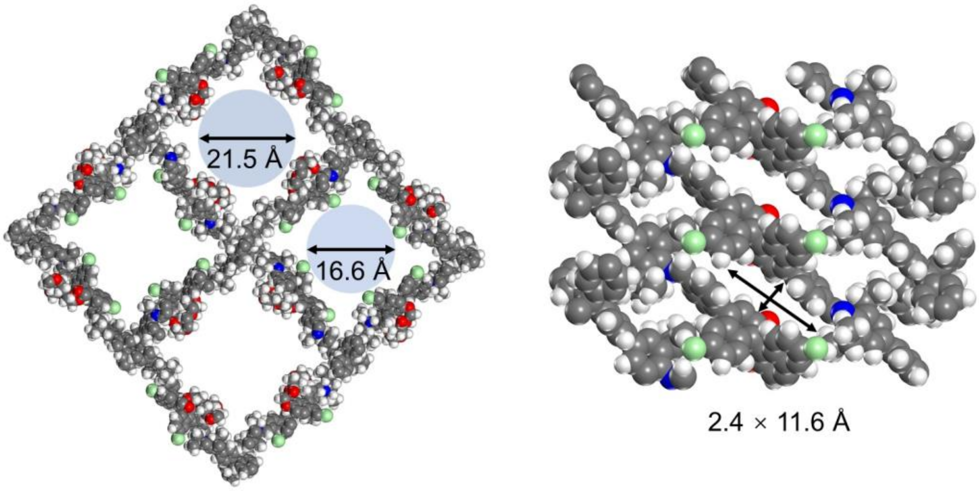

View along the *c*-axis View along the *b*-axis

(b) The pore size of CCOF **31** along *c*-axis and *b*-axis.

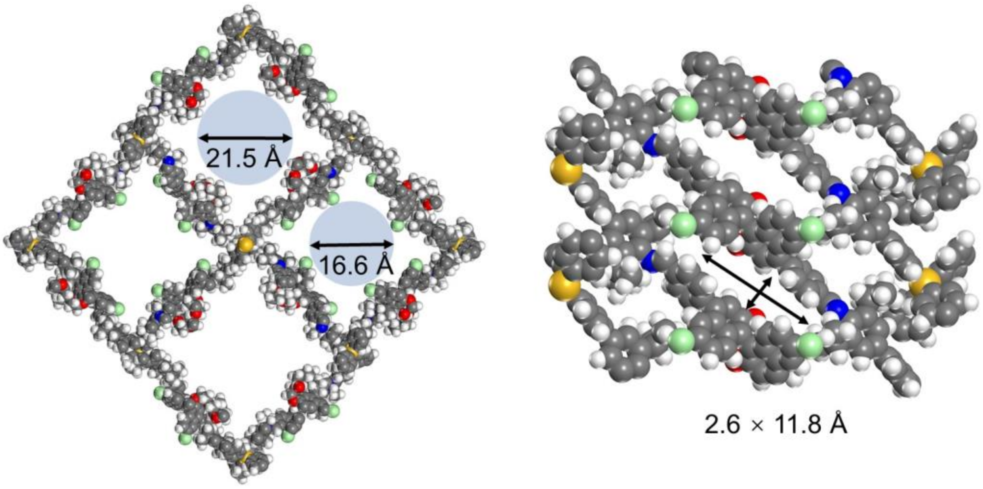

View along the *c*-axis View along the *b*-axis

(c) The dihedral angle of the BDFP-CR6 fragment in CCOF **30**.

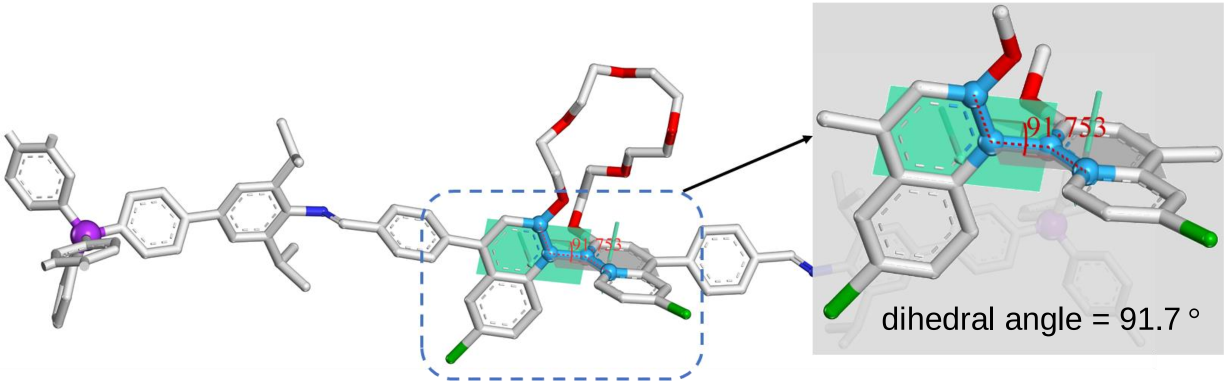

BDFP-CR6 fragment in CCOF **30**

(d) The dihedral angle of the BDFP-CR6 fragment in CCOF **31**.

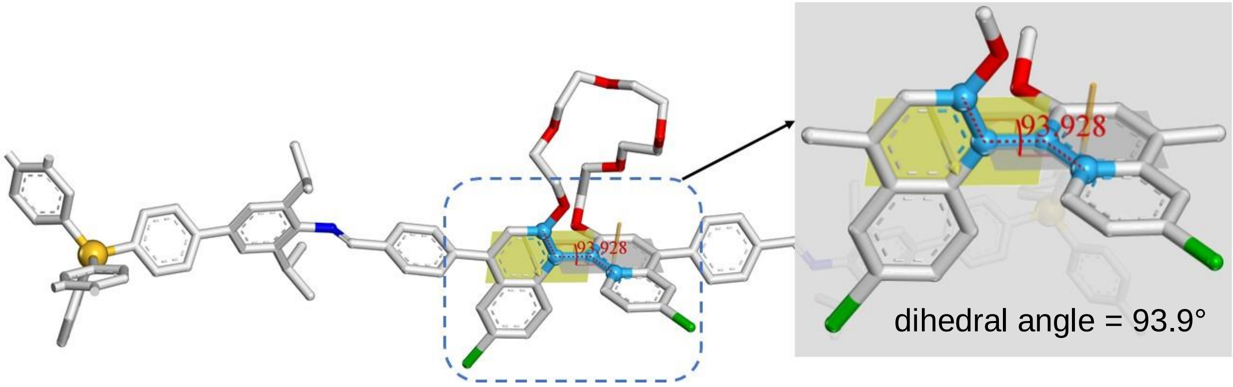

BDFP-CR6 fragment in CCOF **31**

9. Figure S7. TGA curves of CCOFs 30 and CCOF 31.

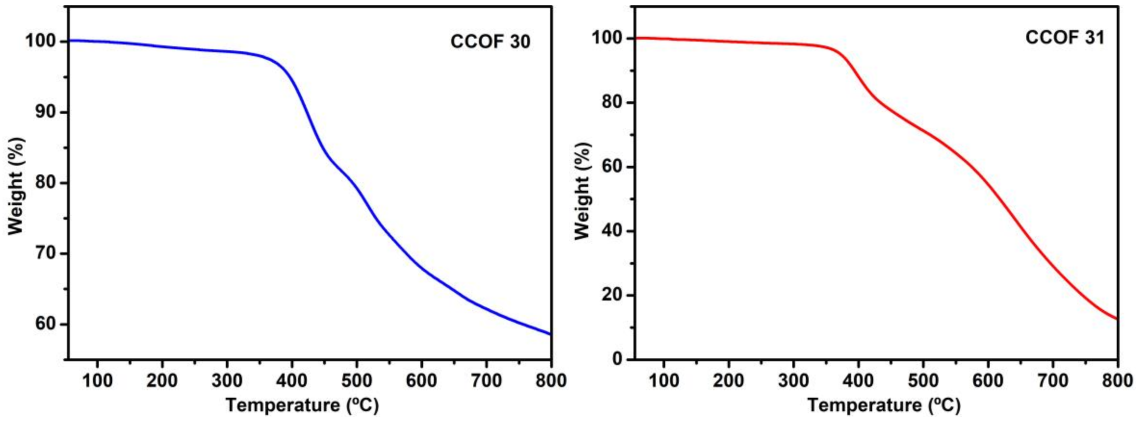

10. Figure S8. BET plots of the CCOFs 30 and 31.

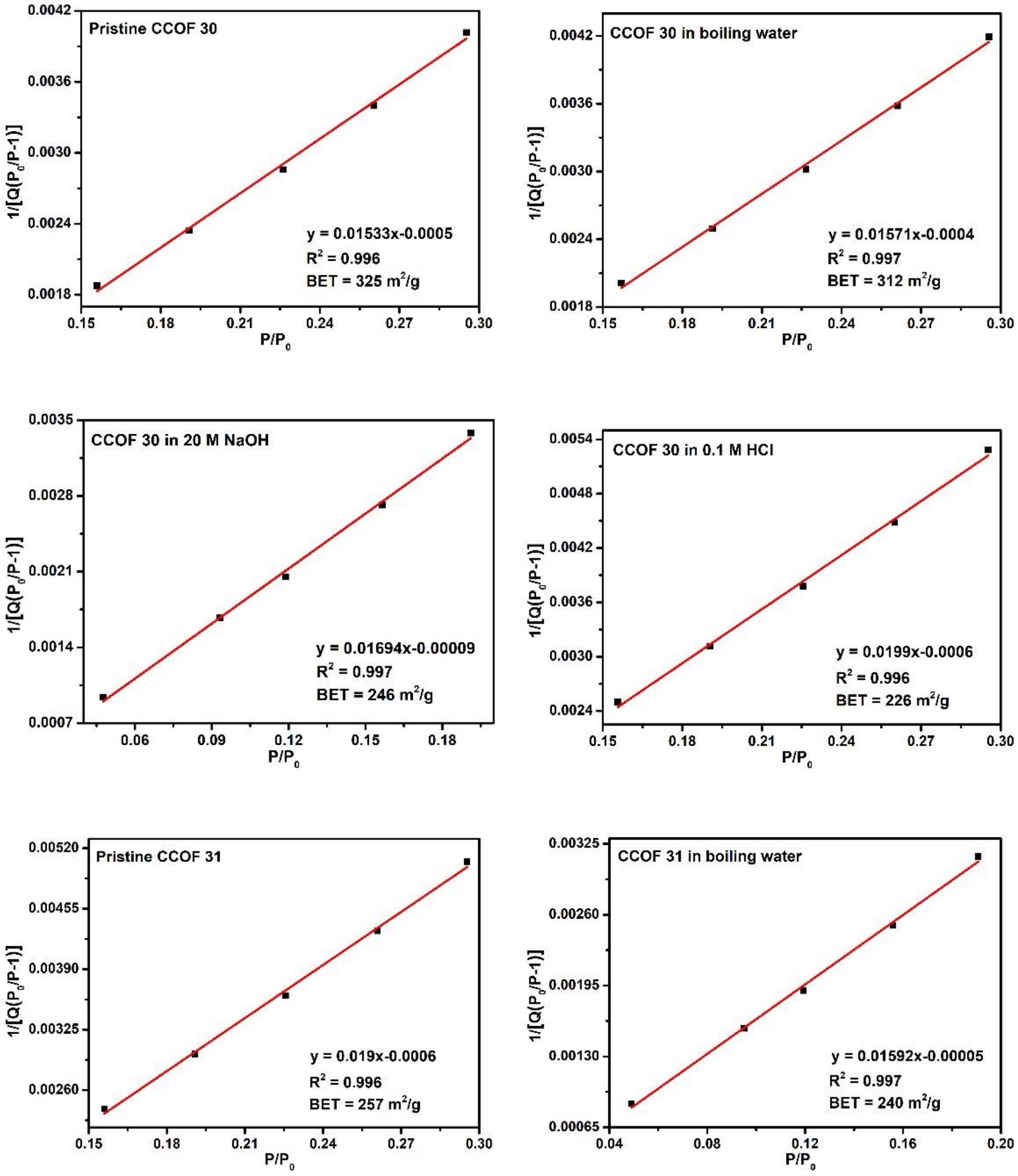

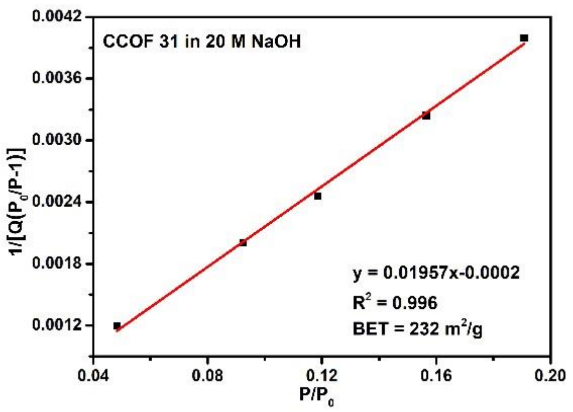

11. Open-tubular Capillary Column Packing

A fuse-silica capillary column (375 μm o.d. × 75 μm i.d.) measuring 60 cm (effective length 52 cm) was swilled with 1 M NaOH for 2.5 h, ultrapure water for 2 h, 0.1 M HCl for 1 h, and then swilled once more with ultrapure water to reach a neutral pH. The column was then dried for 2 h at 120 ℃ in a nitrogen atmosphere. After careful ground the CCOF **30** and CCOF **31** (the particle size is around 0.20-2.24 μm after the crushing process, see below **Figure S9a,b**), then dispersed them in ethanol to prepare the COFs suspension (the particle size is around 0.19-0.69 μm of the COF suspension, see below **Figure S9c,d**). The CCOFs **30** and **31** were coated on the inner wall of the capillary column with a dynamic coating method for electrochromatography experiments.

In addition, the chemical stability of CCOFs **30** and **31** in ethanol was studied by soaking the pristine samples into ethanol for 5 days. The well-maintained crystallinity and porosity, as verified by PXRD patterns and N_2 sorption isotherms, strongly implied that both CCOFs are stable in ethanol. (see below **Figure S10a,b**)

Figure S9. Additional SEM images of (a) CCOF **30** and (b) CCOF **31** after the crushing process. SEM images of the prepared suspension of (c) CCOF **30** and (d) CCOF **31**.

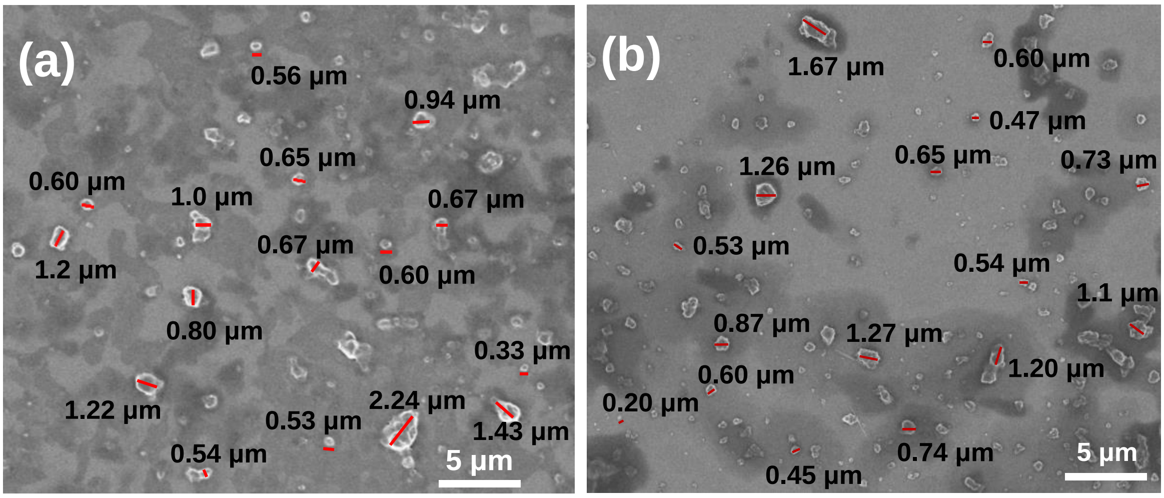

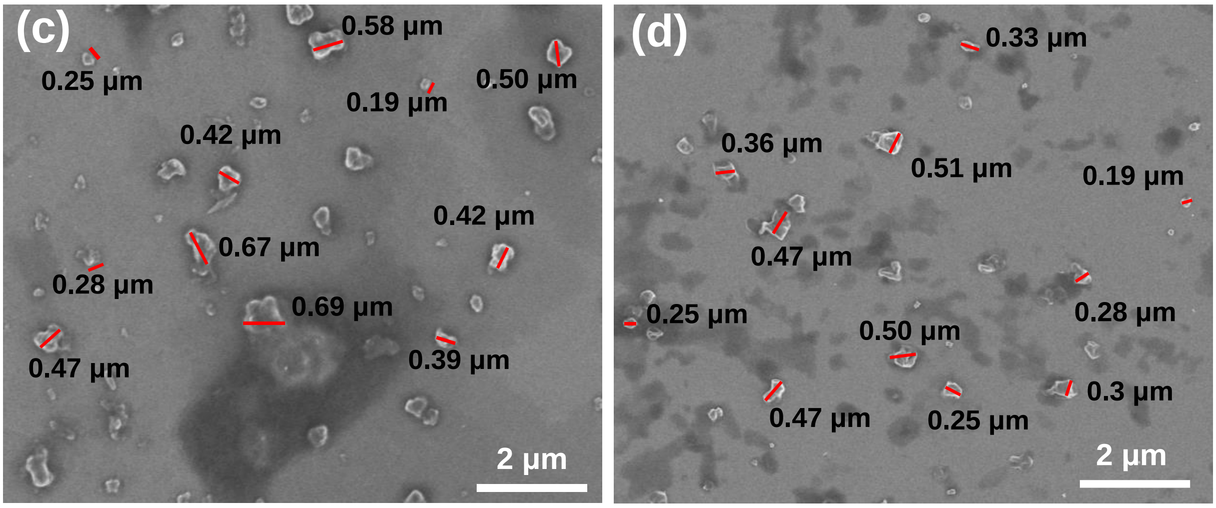

Figure S10. The additional chemical stability of CCOFs 30 and 31.

(a) The additional PXRD patterns of CCOFs **30** and **31** after treated with ethanol; (b) The additional N_2 adsorption isotherms of CCOFs **30** and **31** after treated with ethanol.

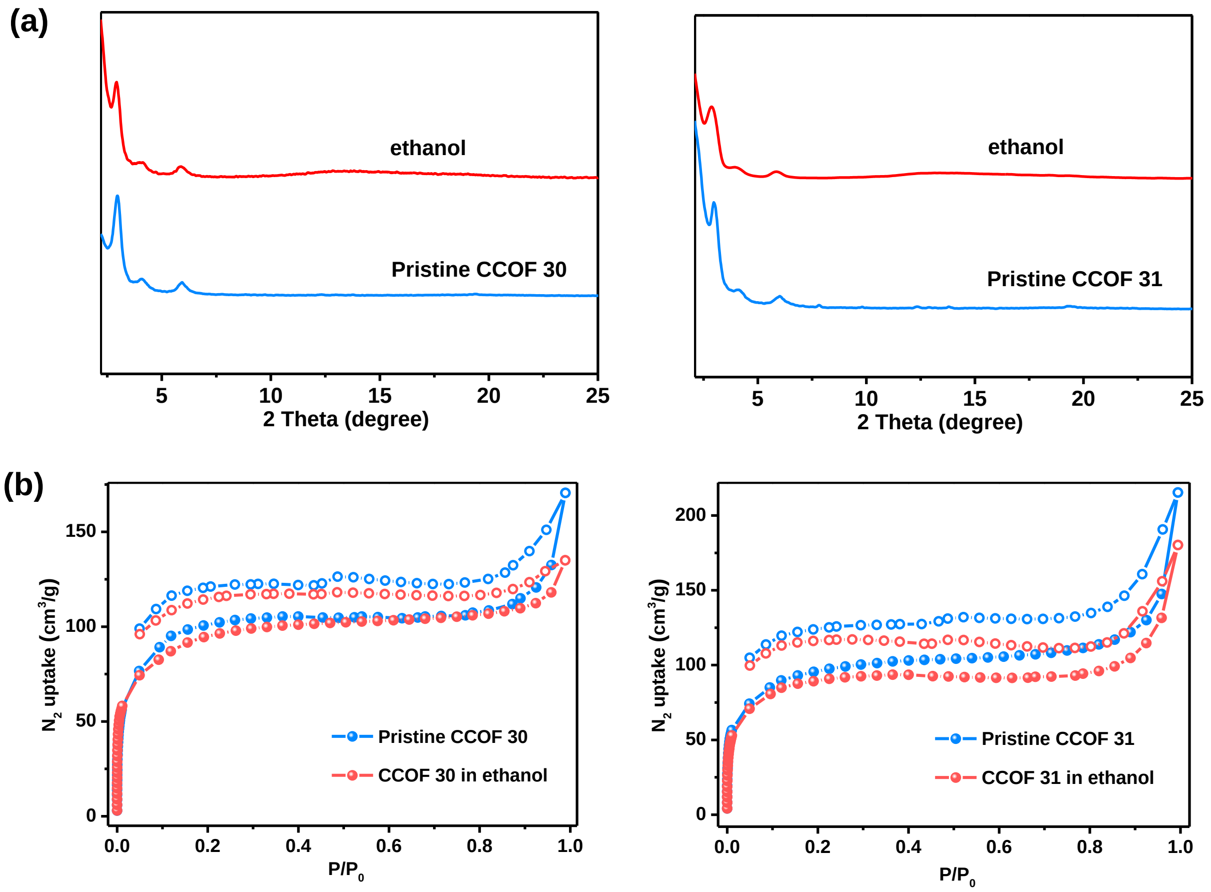

12. CEC procedures

Tris-H_3PO_4 buffer solution (pH = 7.5, 100 mM) was prepared, and it was degassed via ultrasonic bath. Thiourea was used as the indicator of EOF. After washing the coated capillary column with the prepared buffer solution, CEC separation was run until a steady baseline was obtained. The working voltage was 15 kV. During data collection, detection occurred at 254 nm. The injection lasted 5 seconds and was in 10 cm height.

13. Characteristic of the capillary columns

13.1 Figure S11. SEM images of (a) CCOF **30** coated column, and (b) CCOF **31** coated column.

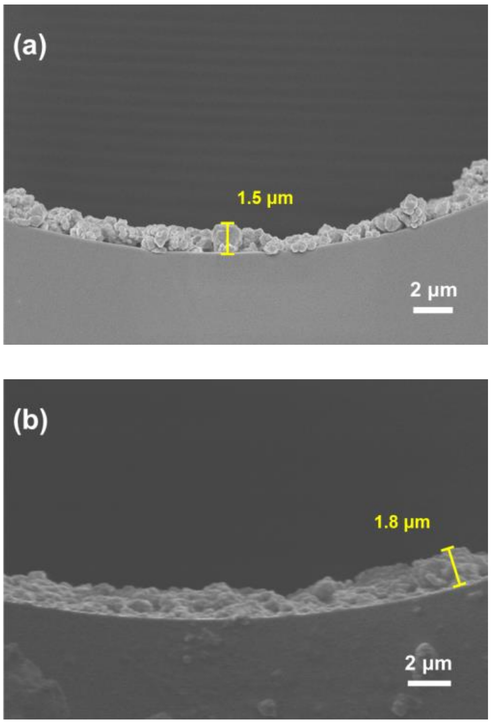

13.2 Current test of the capillary columns.

Figure S12. Effect of the applied voltage on the current of (a) CCOF **30** coated column and (b) CCOF **31** coated column in Tris-H_3PO_4 buffer solution (100 mM, pH = 7.5).

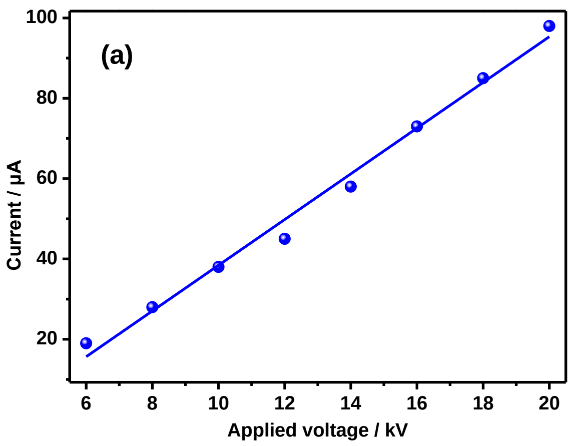

13.3 EOF test of the capillary columns.

Figure S13. Effect of the pH of buffer solution on the EOF of the bare capillary column, (a) CCOF **30** coated column, and (b) CCOF **31** coated column in Tris-H_3PO_4 buffer solution (100 mM) and under 15 kV applied voltage.

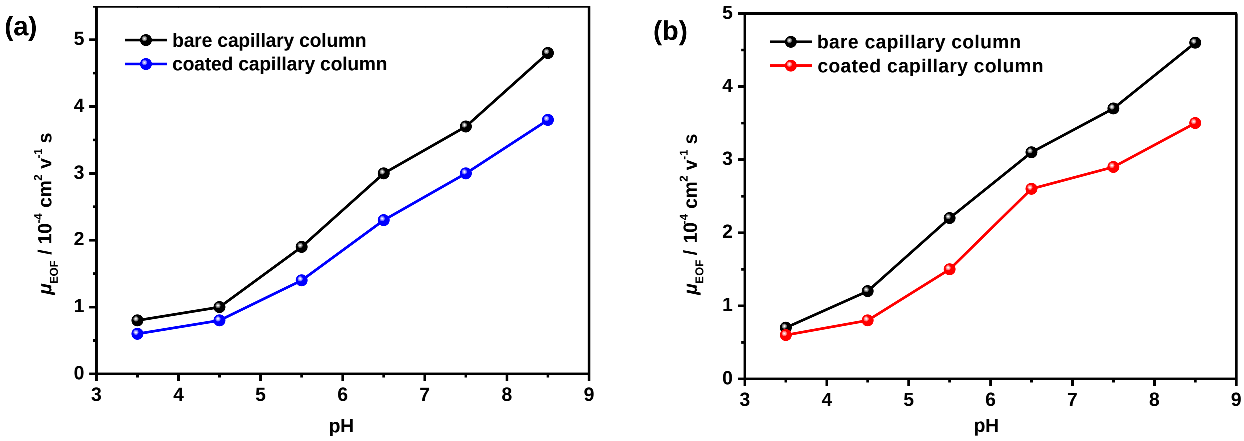

14. Calculation of separation factor and resolution.

The separation factor (α), resolution (Rs), and number of theoretical plate (N) were calculated according to the following equations:

$$\alpha = \frac{t_2}{t_1} \quad (1)$$

$$Rs = 1.18 \times \frac{t_2 - t_1}{W_{1/2(1)} + W_{1/2(2)}} \quad (2)$$

$$N = \frac{5.54}{L} \times \left(\frac{t_R}{W_{1/2}} \right)^2 \qquad (3)$$

15. The CEC chromatograms.

Figure S14. Representative CEC chromatograms on the CCOF **30** coated column and CCOF **31** coated column.

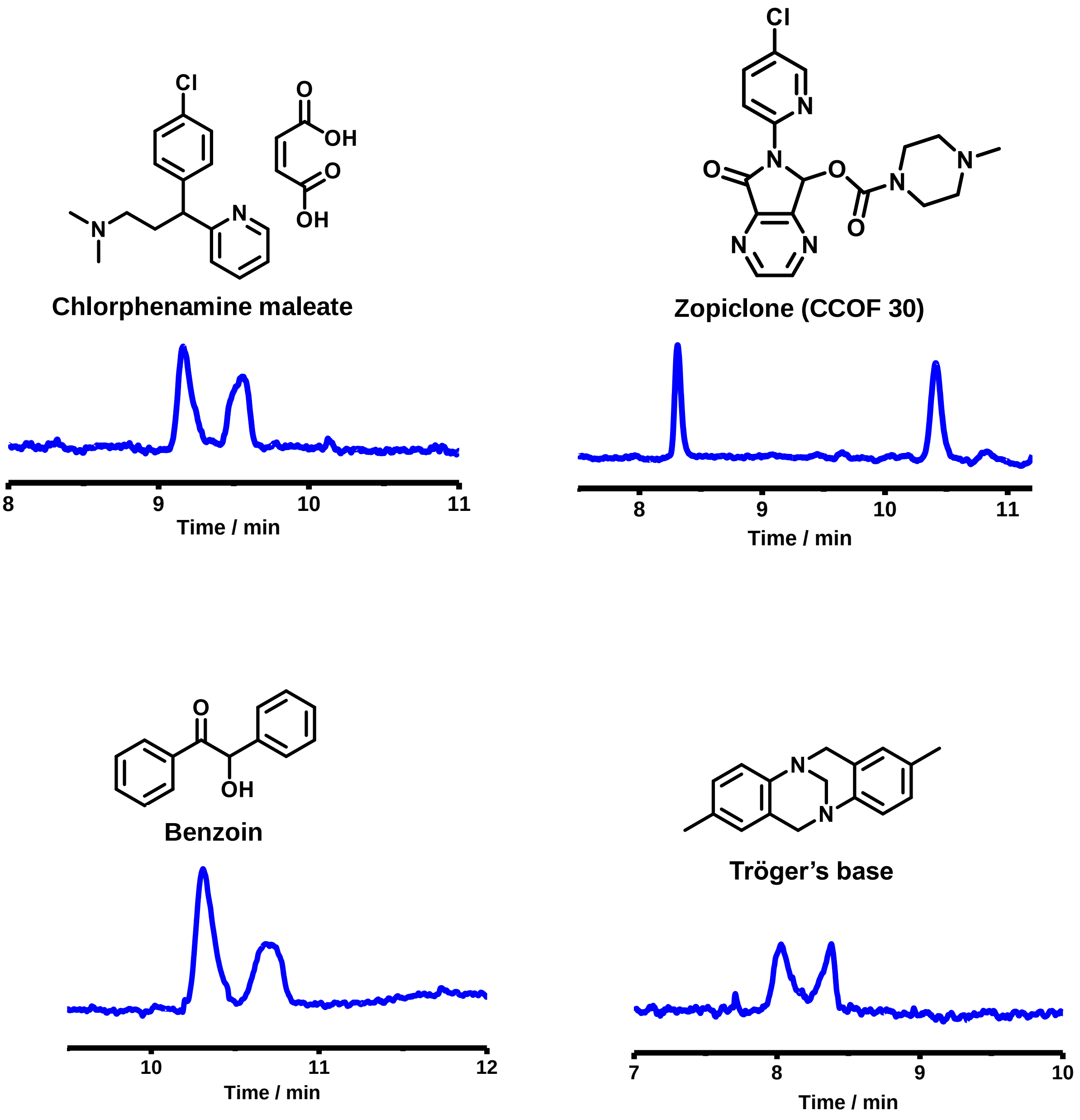

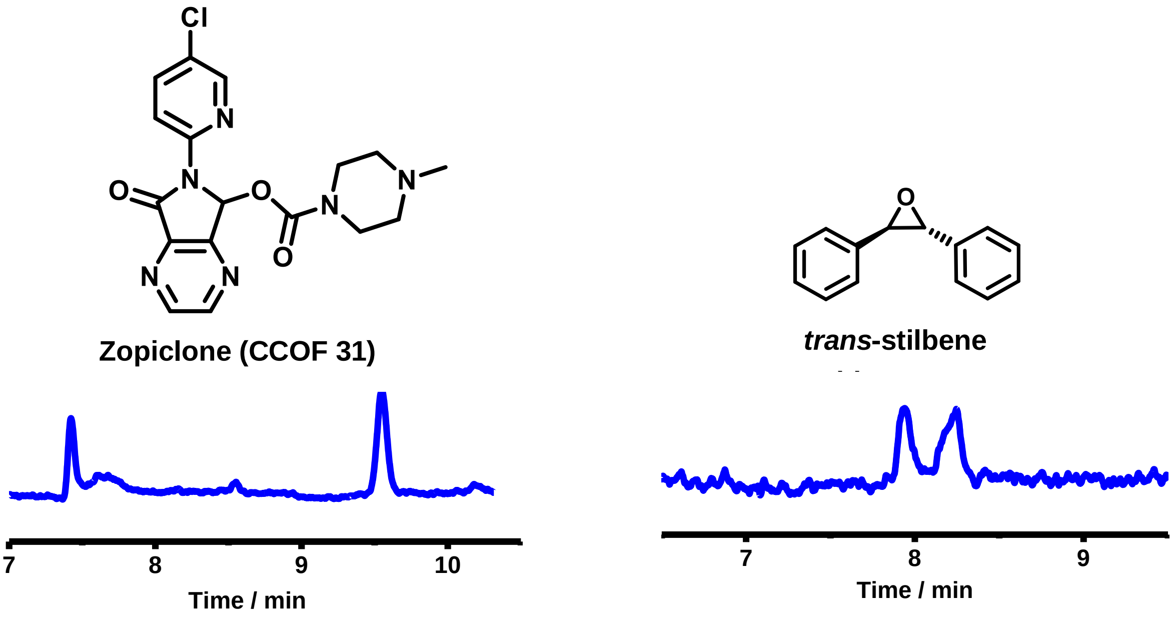

16. The space-filling models of racemates.

Figure S15. The space-filling models of racemates were calculated by Materials Studio 2019 and fully optimized using MS Forcite molecular dynamics module (universal force fields, Ewald summations) method.

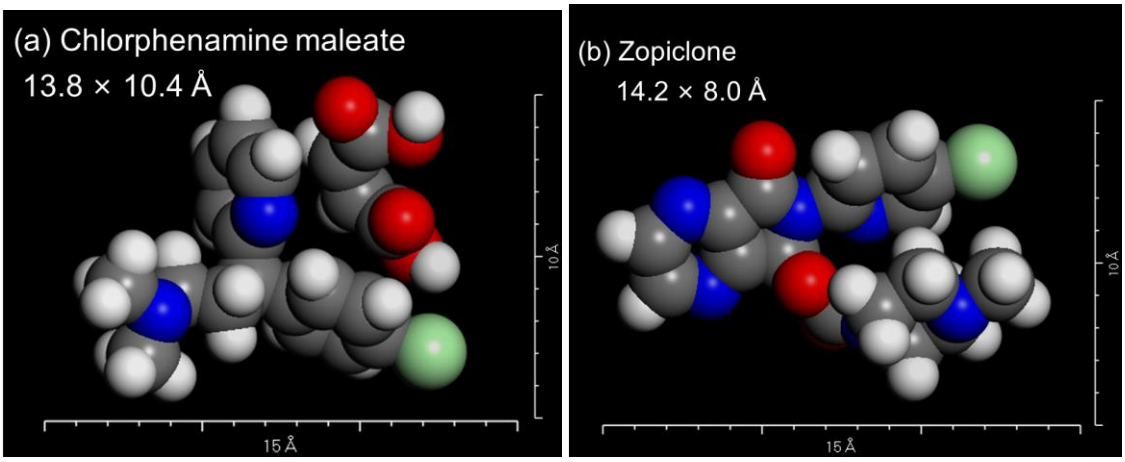

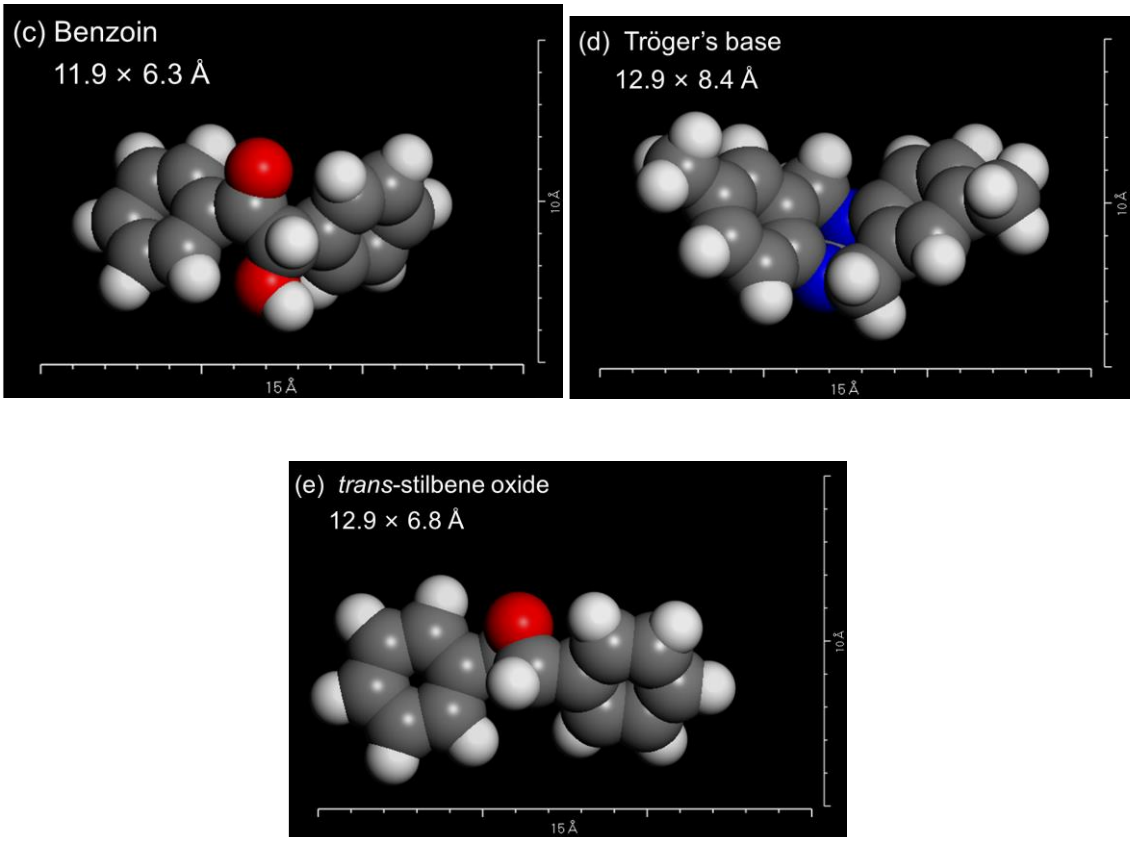

17. The effects of separation conditions.

17.1 Figure S16. Separation chromatograms of enantiomers on CCOFs **30** and **31** coated column under different separation voltages. The chromatographic conditions: capillary column effective length was 52 cm, the Tris-H_3PO_4 buffer solution (pH was 7.5, concentration was 100 mM).

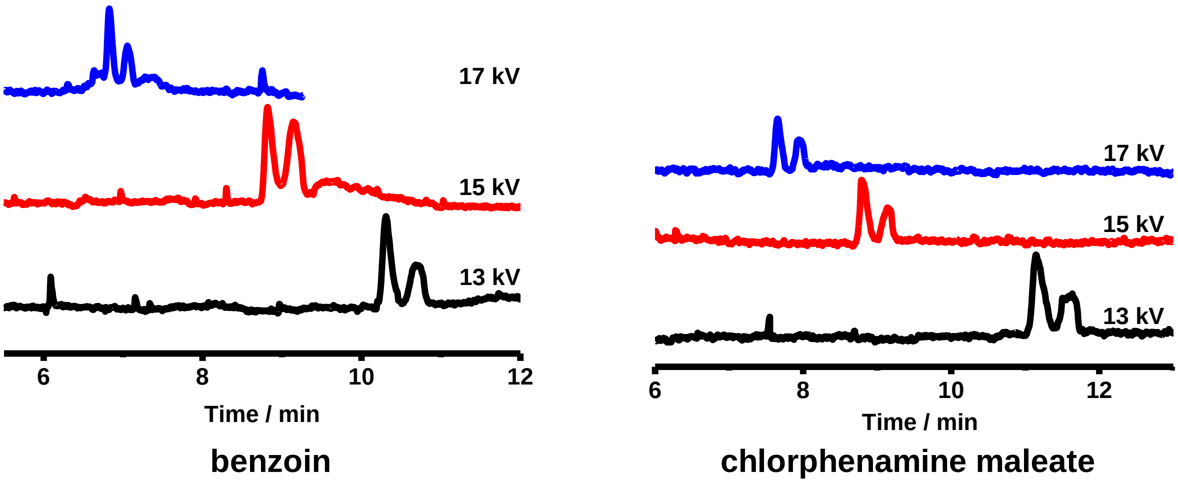

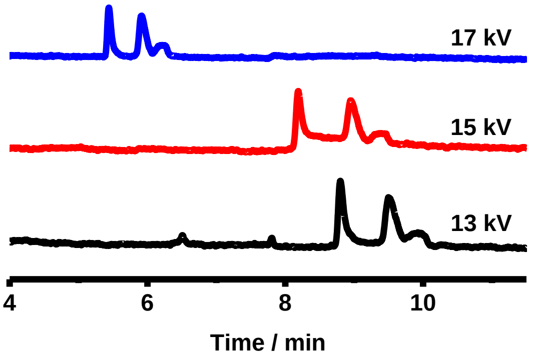

zopiclone (CCOF 30)

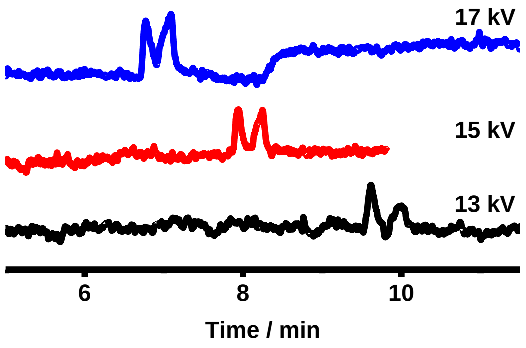

trans-stilbene oxide

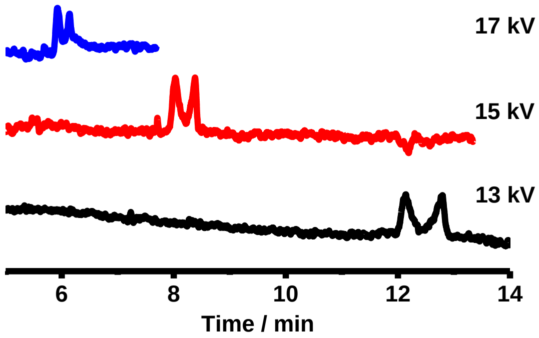

Tröger's base

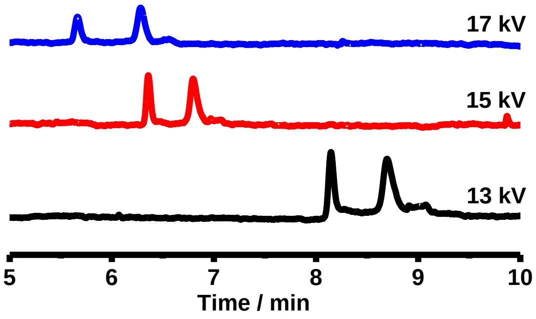

zopiclone (CCOF 31)

17.2 Table S1. CEC enantioseparation data of racemates on CCOFs **30** and **31** coated column under different applied voltages. The chromatographic conditions were the same as the **Figure S16**.

Racemates	Applied voltage (kV)	t_1	t_2	α	*Rs*
benzoin	13	10.31	10.68	1.04	1.42
	15	8.82	9.14	1.04	1.60
	17	6.83	7.05	1.03	1.28
chlorphenamine maleate	13	11.15	11.64	1.04	1.41
	15	8.79	9.14	1.04	1.58
	17	7.66	7.94	1.04	1.53
zopiclone (CCOF **30**)	13	8.80	9.50	1.08	2.74
	15	8.19	8.95	1.09	3.50
	17	5.44	5.91	1.09	2.38
trans-stilbene oxide	13	9.62	10.00	1.04	1.52
	15	7.94	8.25	1.04	1.72
	17	6.77	7.09	1.05	1.43
Tröger's base	13	12.14	12.80	1.05	2.08

	15	8.03	8.38	1.04	2.35
	17	5.93	6.14	1.04	1.45
zopiclone (CCOF **31**)	13	8.15	8.69	1.07	3.60
	15	6.36	6.80	1.07	4.30
	17	5.66	6.28	1.11	3.75

17.3 Figure S17. Enantioseparation chromatograms of racemates on CCOFs **30** and **31** coated capillary columns under different concentrations of Tris-H_3PO_4 buffer solution. The chromatographic conditions: effective length of capillary column was 52 cm, separation voltage was 15 kV, and the pH of buffer solution was 7.5.

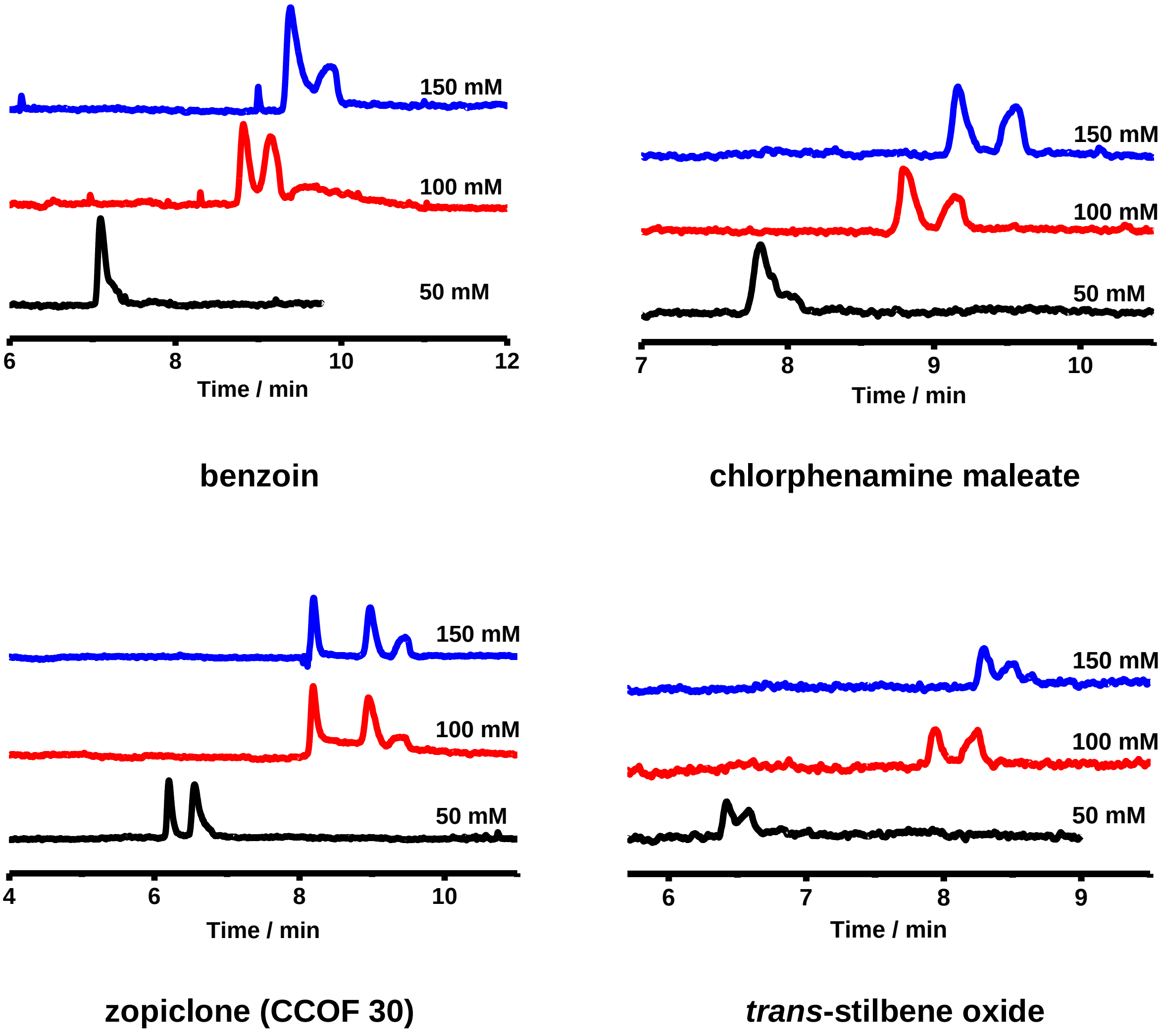

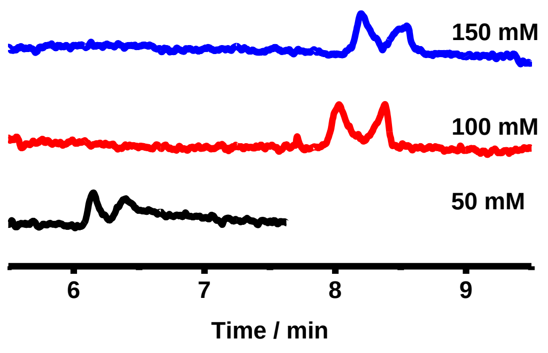

Tröger's base

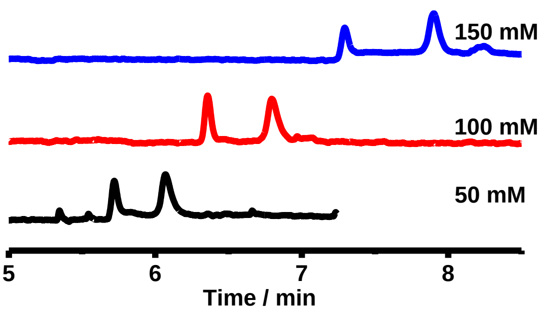

zopiclone (CCOF 31)

17.4 Table S2. Enantioseparation data of racemates on CCOFs **30** and **31** coated capillary columns under different concentrations of Tris-H_3PO_4 buffer solution. The chromatographic conditions were the same as **Figure S17**.

Racemate	Concentration (mM)	t_1	t_2	α	*Rs*
benzoin	50	7.10		1.00	-[a]
	100	8.82	9.14	1.04	1.60
	150	9.39	9.87	1.05	1.55
chlorphenamine maleate	50	7.82		1.00	-[a]
	100	8.79	9.14	1.04	1.58
	150	9.16	9.56	1.04	2.00
zopiclone (CCOF **30**)	50	6.20	6.55	1.06	2.19
	100	8.19	8.95	1.09	3.50
	150	8.19	8.97	1.10	5.38
trans-stilbene oxide	50	6.42	6.58	1.02	1.02
	100	7.94	8.25	1.04	1.72
	150	8.29	8.51	1.03	1.22
Tröger's base	50	6.15	6.40	1.04	1.09
	100	8.03	8.38	1.04	2.35
	150	8.20	8.55	1.04	1.45
zopiclone (CCOF **31**)	50	5.72	6.07	1.06	3.62
	100	6.36	6.80	1.07	4.30
	150	7.29	7.09	1.08	5.10

[a]Can not be separated.

17.5 Figure S18. Enantioseparation chromatograms of racemates on CCOF **30** and **31** coated capillary columns under different pH of Tris-H_3PO_4 buffer solution. The chromatographic conditions: effective length of capillary column was 52 cm, separation voltage was 15 kV, and the concentration of buffer solution for separate chlorphenamine maleate and zopiclone was 150 mM, and the rest of the analytes was 100 mM).

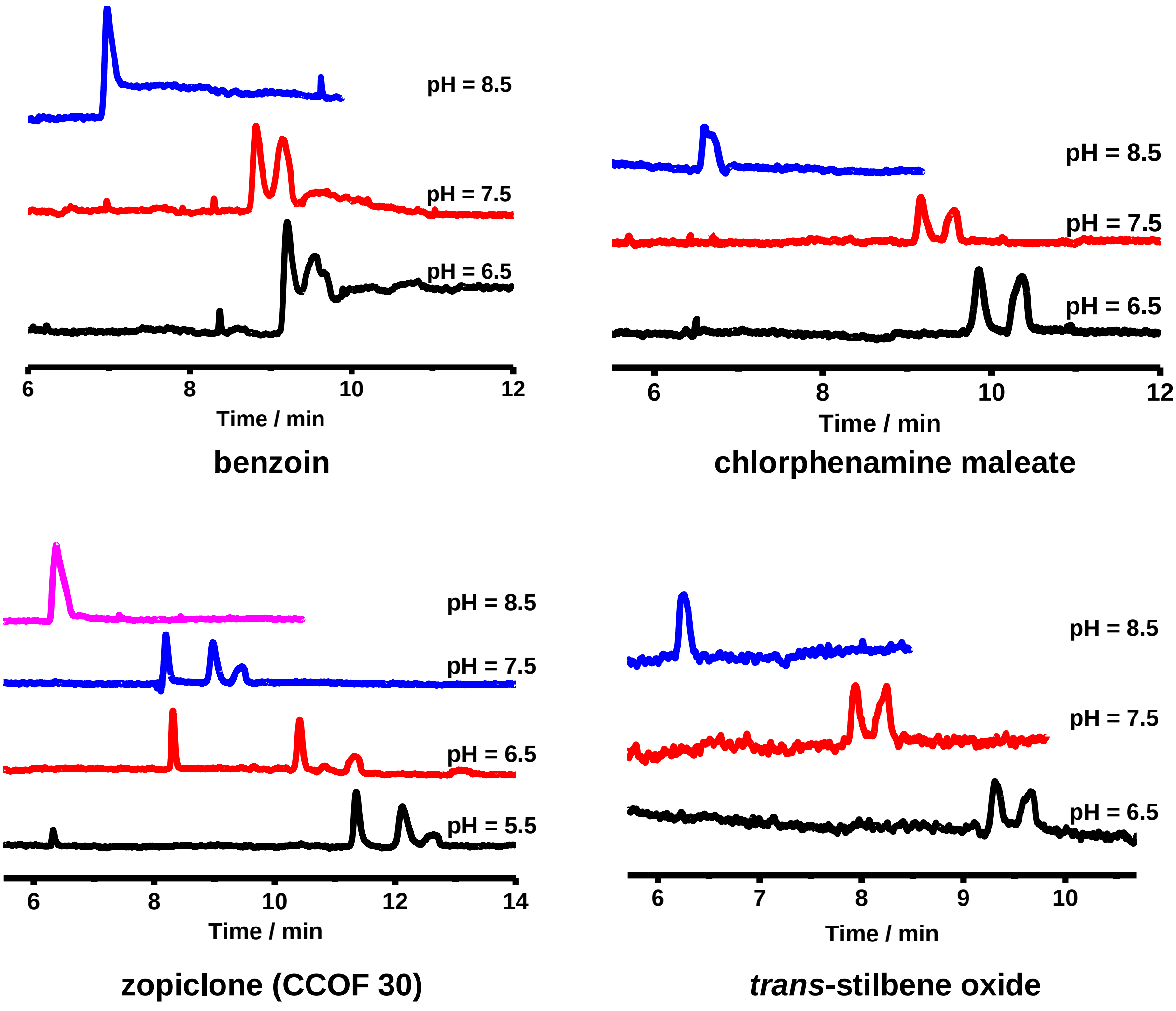

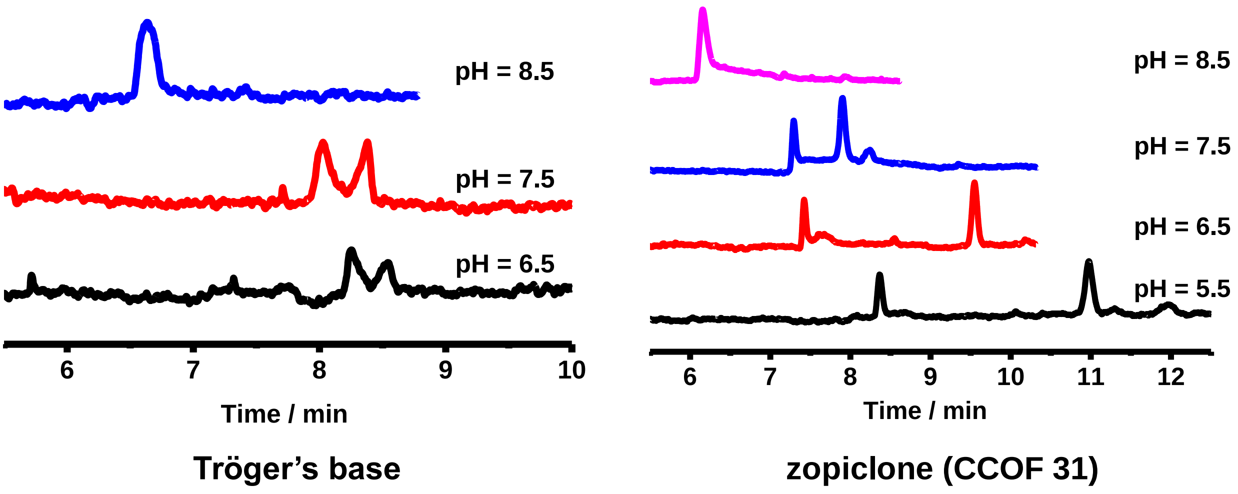

17.6 Table S3. Enantioseparation data of analytes on CCOFs **30** and **31** coated capillary columns under different pH of Tris-H_3PO_4 buffer solution. The chromatographic conditions were the same as **Figure S18**.

Racemate	pH	t_1	t_2	α	*Rs*
benzoin	6.5	9.20	9.55	1.04	1.02
	7.5	8.82	9.14	1.04	1.60
	8.5	6.98		1.00	-[a]
chlorphenamine maleate	6.5	9.85	10.35	1.05	1.89
	7.5	9.16	9.56	1.04	2.00
	8.5	6.60		1.00	-[a]
zopiclone (CCOF **30**)	5.5	11.36	12.12	1.07	3.46
	6.5	8.31	10.41	1.22	16.55
	7.5	8.19	8.97	1.10	5.38
	8.5	6.37		1.00	-[a]
trans-stilbene oxide	6.5	9.31	9.66	1.04	1.61
	7.5	7.94	8.25	1.04	1.72
	8.5	6.26		1.00	-[a]
Tröger's base	6.5	8.25	8.54	1.04	1.47
	7.5	8.03	8.38	1.04	2.35
	8.5	6.63		1.00	-[a]
zopiclone (CCOF **31**)	5.5	8.37	10.98	1.31	16.21
	6.5	7.42	9.55	1.27	20.90
	7.5	7.29	7.09	1.08	5.10
	8.5	6.16		1.00	-[a]

[a]Can not be separated.

18. Repeatability.

Figure S19. CEC chromatograms of (a) chlorphenamine maleate enantiomers on the CCOF **30** coated capillary column and (b) Tröger's base on the CCOF **31** coated capillary column using the same chromatographic conditions obtained for 5 runs.

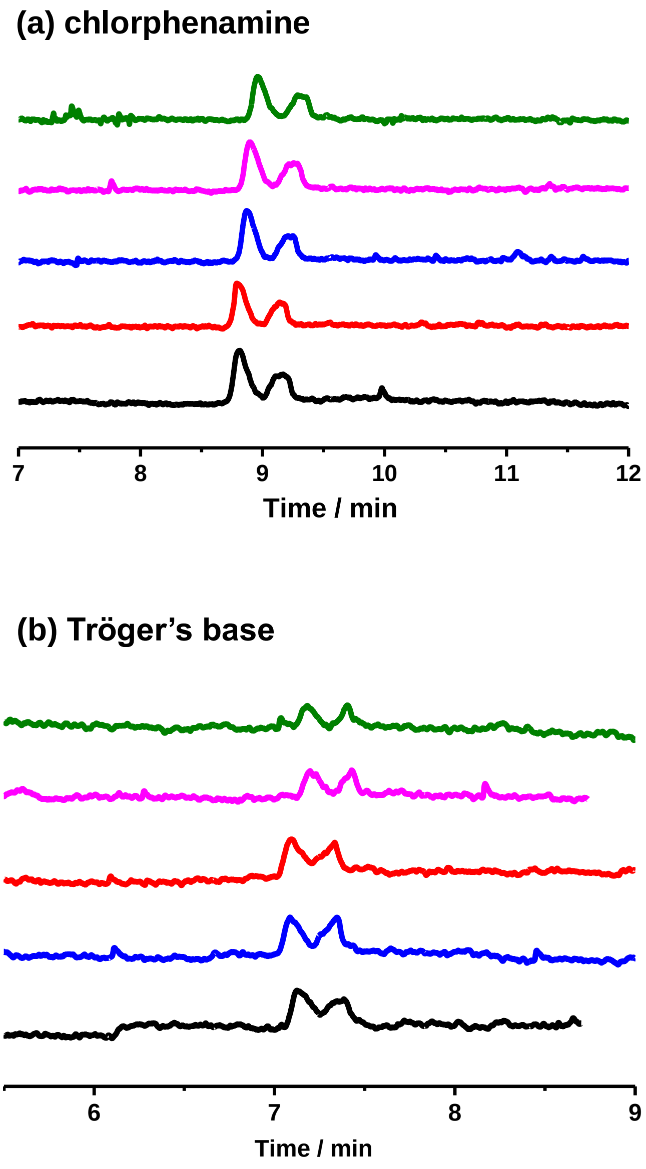

19. Fractional atomic coordinates and unit cell parameters.

Table S4. Fractional atomic coordinates of for the unit cell of CCOF **30**.

CCOF **30**: Space group: ***I*4$_1$**<br>$a = b$ = 60.4533 Å, c = 9.3785 Å<br>$\alpha = \beta = \gamma$ = 90.00 °			
Atom	x	y	z
C1	-0.01571	0.55399	0.48826
C2	0.00544	0.54446	0.48384
C3	0.01007	0.52722	0.38943
C4	-0.00595	0.51943	0.2922
C5	-0.02743	0.52852	0.30171
C6	-0.0322	0.54545	0.39824
C7	-0.02037	0.57322	0.58275
C8	-0.03426	0.59027	0.53582
C9	-0.03815	0.60915	0.6193
C10	-0.02719	0.6113	0.75235
C11	-0.01398	0.59356	0.80484
C12	-0.01065	0.57486	0.718
C13	-0.0535	0.62687	0.55888
C14	-0.07318	0.63168	0.65726
C15	-0.04104	0.64821	0.52433
C16	-0.01237	0.64371	0.86335
N17	-0.02934	0.63144	0.83269
C18	-0.01439	0.66202	0.96476
C19	-0.03263	0.67636	0.963
C20	-0.03416	0.69348	1.06321
C21	-0.01787	0.69583	1.16899
C22	0.00042	0.68155	1.1688
C23	0.00214	0.66478	1.0676

C24	-0.01896	0.71368	1.27712
C25	-0.00064	0.7277	1.28806
C26	0.00087	0.7438	1.39438
C27	-0.01683	0.7467	1.48927
C28	-0.03562	0.73276	1.48165
C29	-0.03667	0.71602	1.37497
C30	-0.05502	0.70165	1.37306
C31	-0.07182	0.70362	1.47353
C32	-0.07106	0.7202	1.57587
C33	-0.05317	0.73479	1.58028
C34	-0.015	0.76426	1.59862
C35	-0.02332	0.78578	1.57291
C36	-0.02063	0.80247	1.6781
C37	-0.00984	0.79728	1.80756
C38	-0.00187	0.77588	1.83102
C39	-0.0046	0.75914	1.72861
C40	-0.02831	0.82405	1.64918
C41	-0.03852	0.82898	1.52012
C42	-0.04188	0.81248	1.41922
C43	-0.03425	0.79105	1.44417
O44	0.00305	0.73723	1.74908
O45	0.01942	0.75792	1.40458
C46	-0.00648	0.81385	1.921
C47	0.01485	0.82163	1.95109
C48	0.01804	0.83758	2.0569
C49	-0.00006	0.84563	2.13508
C50	-0.02133	0.8376	2.10602
C51	-0.02454	0.82174	1.99966
C52	0.00275	0.86308	2.24249
C53	0.01493	0.91622	2.28436

C54	0.02128	0.89484	2.33238
N55	0.01966	0.87631	2.23767
C56	0.02768	0.89156	2.47651
C57	0.02659	0.90945	2.57179
C58	0.01919	0.9302	2.52699
C59	0.01385	0.93358	2.38362
C60	0.01506	0.94783	2.63236
C61	-0.00578	0.95777	2.63833
C62	-0.01027	0.97387	2.74021
C63	0.00579	0.98058	2.84049
C64	0.02697	0.97069	2.83128
C65	0.03144	0.95439	2.72918
C66	-0.00363	0.59411	0.95398
C67	0.02169	0.59471	0.94572
C68	-0.01078	0.57427	1.04406
C69	0.00859	0.92126	2.12982
C70	0.03459	0.86883	2.53179
C71	-0.01249	0.90931	2.08188
C72	0.02751	0.91641	2.02632
C73	0.05755	0.86936	2.60099
C74	0.01762	0.85996	2.63868
C75	0.04096	0.7481	1.38893
C76	0.04534	0.73062	1.50352
O77	0.03709	0.70972	1.45889
C78	0.0362	0.69553	1.57926
C79	0.02871	0.67252	1.5345
O80	0.0296	0.65779	1.65225
C81	0.05088	0.64799	1.66749
C82	0.06021	0.65296	1.81559
O83	0.07073	0.67394	1.81369

C84	0.06844	0.68512	1.94577
C85	0.04663	0.69801	1.95388
O86	0.04777	0.71731	1.86757
C87	0.03367	0.73427	1.91954
C88	0.009	0.72983	1.88923
Cl89	-0.09337	0.68438	1.47516
Cl90	-0.04633	0.85622	1.4825
H91	0.0186	0.55061	0.55137
H92	0.02645	0.52017	0.38942
H93	-0.04085	0.52253	0.23659
H94	-0.04894	0.5518	0.40306
H95	-0.0416	0.5892	0.43077
H96	-0.00055	0.56139	0.75691
H97	-0.06068	0.62082	0.45613
H98	-0.08204	0.61613	0.68435
H99	-0.08516	0.64294	0.60356
H100	-0.06799	0.63983	0.75772
H101	-0.02618	0.64469	0.45844
H102	-0.03583	0.65716	0.62188
H103	-0.05206	0.6593	0.46308
H104	0.00402	0.63903	0.82765
H105	-0.04547	0.67436	0.88341
H106	-0.04807	0.70478	1.05918
H107	0.01338	0.68349	1.24746
H108	0.01619	0.65368	1.07108
H109	0.01275	0.72572	1.21384
H110	-0.05645	0.68837	1.2969
H111	-0.08436	0.72143	1.6527
H112	-0.05301	0.74734	1.66274
H113	0.00658	0.77259	1.93018

H114	-0.02626	0.8374	1.72461
H115	-0.05034	0.81644	1.32104
H116	-0.03688	0.77876	1.36187
H117	0.02891	0.81554	1.89117
H118	0.03455	0.84372	2.07796
H119	-0.03549	0.84406	2.16362
H120	-0.04108	0.81575	1.97723
H121	-0.01065	0.86622	2.31702
H122	0.03047	0.90691	2.68305
H123	0.00827	0.94981	2.3504
H124	-0.0187	0.95286	2.56469
H125	-0.02641	0.98135	2.74067
H126	0.04042	0.97564	2.89975
H127	0.04771	0.94694	2.72494
H128	-0.00936	0.60901	1.01335
H129	0.02732	0.60902	0.8811
H130	0.02829	0.57941	0.89592
H131	0.02888	0.59627	1.05432
H132	-0.0057	0.57655	1.15709
H133	-0.00289	0.55901	1.00431
H134	-0.02905	0.57225	1.04107
H135	0.00499	0.93924	2.12057
H136	0.03549	0.85688	2.44114
H137	-0.00908	0.89197	2.05053
H138	-0.01962	0.91762	1.98677
H139	-0.02512	0.90938	2.16807
H140	0.02254	0.92117	1.91683
H141	0.03184	0.89856	2.02534
H142	0.04244	0.92607	2.05627
H143	0.06986	0.87635	2.52533

H144	0.06286	0.85231	2.62924
H145	0.05765	0.87946	2.69986
H146	0.02113	0.84235	2.66408
H147	0.00071	0.86095	2.5923
H148	0.01774	0.86958	2.73957
H149	0.05338	0.76151	1.40156
H150	0.04371	0.74108	1.2813
H151	0.0375	0.7367	1.60403
H152	0.06351	0.72943	1.5215
H153	0.02417	0.70165	1.6601
H154	0.05262	0.69415	1.63186
H155	0.03827	0.66667	1.44016
H156	0.01122	0.67382	1.49871
H157	0.06344	0.65328	1.58712
H158	0.04897	0.62979	1.6563
H159	0.07276	0.64019	1.8427
H160	0.04645	0.65152	1.89402
H161	0.08213	0.6973	1.95556
H162	0.07002	0.67364	2.03831
H163	0.04348	0.70175	2.06851
H164	0.03323	0.6871	1.91421
H165	0.03834	0.74993	1.86539
H166	0.03646	0.73749	2.03459
H167	-0.00172	0.73737	1.97232
H168	0.00544	0.71188	1.89649
C169	0	0	-0.05893

Table S5. Fractional atomic coordinates of for the unit cell of CCOF **31**.

CCOF **31**: Space group: ***I*4$_1$**<br>*a* = *b* = 60.9056 Å, *c* = 9.6507 Å<br>*α* = *β* = *γ* = 90.00 °			
Atom	x	y	z
C1	-0.01817	0.55665	0.48116
C2	0.00238	0.5463	0.48892
C3	0.00796	0.52984	0.39442
C4	-0.00697	0.52337	0.29168
C5	-0.02768	0.53341	0.28546
C6	-0.03317	0.55004	0.37903
C7	-0.02356	0.57493	0.57606
C8	-0.03082	0.59495	0.52245
C9	-0.0344	0.61314	0.60902
C10	-0.03015	0.61113	0.75329
C11	-0.02538	0.59009	0.81009
C12	-0.02144	0.57241	0.71942
C13	-0.04232	0.63439	0.54196
C14	-0.06369	0.6427	0.60723
C15	-0.02462	0.65215	0.54568
C16	-0.01369	0.63627	0.91473
N17	-0.03046	0.6304	0.8399
C18	-0.01469	0.65551	1.0073
C19	-0.03046	0.67211	0.98929
C20	-0.03124	0.68997	1.08061
C21	-0.01656	0.69121	1.19259
C22	-0.00039	0.67501	1.20741
C23	0.00044	0.65722	1.11629
C24	-0.01792	0.70931	1.2953

C25	0.00045	0.72292	1.31148
C26	0.00079	0.73989	1.40973
C27	-0.01766	0.74336	1.49412
C28	-0.03653	0.72999	1.47946
C29	-0.0367	0.71283	1.37928
C30	-0.05558	0.69949	1.36777
C31	-0.07399	0.70342	1.44931
C32	-0.07393	0.72027	1.54618
C33	-0.05535	0.73344	1.5624
C34	-0.01692	0.76151	1.59672
C35	-0.02533	0.78255	1.56382
C36	-0.02441	0.79963	1.66402
C37	-0.01507	0.79539	1.79545
C38	-0.00724	0.77436	1.82669
C39	-0.00796	0.7573	1.72859
C40	-0.03245	0.82063	1.62871
C41	-0.04095	0.82471	1.49724
C42	-0.04221	0.80793	1.4001
C43	-0.03447	0.78699	1.43224
O44	0.00033	0.73599	1.75535
O45	0.01839	0.75495	1.41821
C46	-0.01207	0.8128	1.90051
C47	0.00887	0.82162	1.92316
C48	0.012	0.83792	2.02318
C49	-0.00576	0.84544	2.10301
C50	-0.02671	0.83653	2.08045
C51	-0.02987	0.82028	1.97966
C52	-0.00282	0.86283	2.20775
C53	0.01496	0.91455	2.25894
C54	0.01755	0.89256	2.30587

N55	0.01495	0.87467	2.2104
C56	0.02193	0.88833	2.44801
C57	0.02172	0.90589	2.54247
C58	0.01733	0.92728	2.49857
C59	0.01448	0.93156	2.3573
C60	0.01396	0.94489	2.60159
C61	-0.00552	0.95709	2.59847
C62	-0.00954	0.97301	2.69872
C63	0.00577	0.97702	2.80414
C64	0.02547	0.96511	2.80687
C65	0.02951	0.94907	2.70592
C66	-0.02639	0.58584	0.96607
C67	-0.00476	0.57659	1.02263
C68	-0.04535	0.57028	1.00095
C69	0.01264	0.92072	2.10585
C70	0.02666	0.86518	2.50088
C71	-0.00888	0.91258	2.042
C72	0.03221	0.91274	2.01997
C73	0.04948	0.86367	2.56705
C74	0.00929	0.85781	2.60566
C75	0.04038	0.74653	1.40977
C76	0.04534	0.73066	1.52856
O77	0.03861	0.70921	1.49097
C78	0.04008	0.6953	1.6084
C79	0.02832	0.67386	1.57656
O80	0.02457	0.66132	1.69813
C81	0.04386	0.65026	1.74275
C82	0.05114	0.65918	1.88348
O83	0.06337	0.67869	1.86401
C84	0.06608	0.69024	1.99036

C85	0.047	0.70606	2.0189
O86	0.04494	0.72143	1.90922
C87	0.02848	0.73723	1.93898
C88	0.00547	0.72938	1.89451
Cl89	-0.09721	0.68687	1.43074
Cl90	-0.04924	0.85124	1.45166
H91	0.01419	0.55123	0.56693
H92	0.02381	0.52195	0.40254
H93	-0.03972	0.52834	0.20942
H94	-0.04923	0.55763	0.37313
H95	-0.03297	0.59655	0.41145
H96	-0.01722	0.55643	0.76046
H97	-0.04602	0.63123	0.43022
H98	-0.07627	0.62948	0.60883
H99	-0.07049	0.65651	0.54579
H100	-0.06126	0.64865	0.71465
H101	-0.00923	0.64568	0.49933
H102	-0.0209	0.65761	0.65296
H103	-0.02984	0.6667	0.48511
H104	0.00122	0.62654	0.91332
H105	-0.04209	0.67118	0.90469
H106	-0.04351	0.70264	1.06566
H107	0.01142	0.67604	1.29076
H108	0.01272	0.6446	1.13196
H109	0.01446	0.72036	1.2455
H110	-0.05643	0.68581	1.29691
H111	-0.08843	0.72298	1.60868
H112	-0.05589	0.74632	1.63972
H113	-0.00029	0.77167	1.92814
H114	-0.03201	0.83415	1.70119

H115	-0.04914	0.81129	1.29926
H116	-0.03553	0.77446	1.35295
H117	0.02273	0.81591	1.86263
H118	0.02828	0.84465	2.03894
H119	-0.04064	0.84235	2.14026
H120	-0.04612	0.81353	1.963
H121	-0.01631	0.86655	2.2776
H122	0.02406	0.90276	2.65183
H123	0.0115	0.9483	2.32449
H124	-0.01772	0.95408	2.5189
H125	-0.02447	0.98259	2.6924
H126	0.03774	0.96829	2.88554
H127	0.04477	0.93995	2.7084
H128	-0.02995	0.6014	1.02254
H129	0.00905	0.58783	0.99772
H130	-0.00101	0.56021	0.97837
H131	-0.00567	0.57483	1.13722
H132	-0.04702	0.56848	1.11483
H133	-0.0429	0.55381	0.95488
H134	-0.06108	0.577	0.96049
H135	0.01238	0.93897	2.09665
H136	0.02637	0.85333	2.41261
H137	-0.00851	0.89464	2.02311
H138	-0.01166	0.9207	1.94032
H139	-0.02296	0.91647	2.11104
H140	0.03065	0.91847	1.91151
H141	0.03318	0.89455	2.01779
H142	0.04777	0.91927	2.06366
H143	0.06219	0.8692	2.49214
H144	0.05314	0.8464	2.59625

H145	0.05066	0.87399	2.66166
H146	0.01126	0.84006	2.62896
H147	-0.00742	0.86043	2.56216
H148	0.01049	0.86707	2.70444
H149	0.05177	0.76071	1.41848
H150	0.04388	0.73881	1.30822
H151	0.03657	0.73695	1.62224
H152	0.06332	0.73083	1.54947
H153	0.03201	0.70228	1.70232
H154	0.05753	0.69188	1.63355
H155	0.03703	0.66445	1.49435
H156	0.01213	0.67859	1.53315
H157	0.05801	0.65108	1.66944
H158	0.03976	0.63259	1.75497
H159	0.0616	0.64667	1.93549
H160	0.03613	0.6619	1.94631
H161	0.08121	0.70034	1.98162
H162	0.06854	0.67905	2.08052
H163	0.05075	0.71452	2.11822
H164	0.03171	0.69642	2.03243
H165	0.03259	0.75236	1.88025
H166	0.02832	0.74226	2.04998
H167	-0.00733	0.73507	1.96849
H168	0.0049	0.71117	1.8986
Si169	0	0	-0.07444

20. References.

(1) Rivero-Crespo, M. A.; Toupalas, G.; Morandi, B. Preparation of Recyclable and Versatile Porous Poly(Aryl Thioether)s by Reversible Pd-Catalyzed C–S/C–S Metathesis. *J. Am. Chem. Soc.* **2021,** *143,* 21331-21339.

(2) Liu, Y.; Li, W.; Yuan, C.; Jia, L.; Liu, Y.; Huang, A.; Cui, Y. Two-Dimensional Fluorinated Covalent Organic Frameworks with Tunable Hydrophobicity for Ultrafast Oil–Water Separation. *Angew. Chem. Int. Ed.* **2022,** *61,* e202113348.

(3) Kohlhaas, M.; Zähres, M.; Mayer, C.; Engeser, M.; Merten, C.; Niemeyer, J. Chiral Hydrogen-Bonded Supramolecular Capsules: Synthesis, Characterization and Complexation of C_{70}. *Chem. Commun.* **2019,** *55,* 3298-3301.

(4) Talapaneni, S. N.; Buyukcakir, O.; Je, S. H.; Srinivasan, S.; Seo, Y.; Polychronopoulou, K.; Coskun, A. Nanoporous Polymers Incorporating Sterically Confined *N*-Heterocyclic Carbenes for Simultaneous CO_2 Capture and Conversion at Ambient Pressure. *Chem. Mater.* **2015,** *27,* 6818-6826.
